# Supplementary material for: MicroRNA exporter HuR clears the internalized pathogens by promoting pro‐inflammatory response in infected macrophages
Source: EMBO Mol Med. 2020 Feb 7;12(3):e11011. doi: 10.15252/emmm.201911011 (PMC7059013; doi:10.15252/emmm.201911011)
Supplement: Supplementary file 1 — Appendix [file EMMM-12-e11011-s001.pdf]

Appendix of

MicroRNA Exporter HuR Clears the Internalized Pathogens by Promoting Pro-inflammatory Response in Infected Macrophages

Goswami et al.

Table of content

1. **Appendix Table S1** HuR target gene status in *L. donovani* infected (LDI) macrophages **Page 2**
2. **Appendix Table S2** List of experiments and P values **Page 6**
3. **Appendix Table S3** List of Primers used for mRNA detection **Page 11**
4. **Appendix Table S4** List of antibodies used **Page 12**
5. **Appendix Figure S1** Expression profile of HuR targets in *Ld* infected macrophages **Page 13**

Appendix Table 1: HuR target gene status in *L. donovani* infected (LDI) macrophages. HuR target genes found to be differentially expressed in monocyte-derived macrophages exposed to the intracellular protozoan parasite *L. donovani* for 16 hours have been tabulated here.

	Gene Name	log Fold Change (LDI macrophage)	p-value (LDI macrophage)	No. of HuR sites (Lebedeva et al., 2011)	No. of HuR sites (Mukherjee et al., 2011)	HuR (*Elavl1*) knockout murine bone marrow-derived macrophages (Lu et al, 2014)
HuR target genes that show down-regulation upon HuR knockdown in other cell types and are down-regulated in *L. donovani* infection in macrophages	ANGEL2	-1.65	4.80E-04		9	Downregulated
	ANGPT2	-2.24	6.69E-04		1	Upregulated
	CD47	-1.58	1.63E-02		7	Upregulated
	CDC42BPA	-1.56	1.14E-03	14		Downregulated
	CELF1	-3.89	2.98E-04	15		Upregulated
	CYB5A	-2.02	7.43E-03	2		Downregulated
	DIAPH2	-1.77	9.52E-04	9		Downregulated
	ELK4	-2.2	6.88E-04		25	Upregulated
	FASTKD2	-2.21	6.85E-04	2		Downregulated
	FBXL4	-2.61	5.35E-04		10	Upregulated
	GOLGB1	-2.41	3.52E-02	2		Not found
	KIF3A	-3.11	4.14E-04		23	Upregulated
	MSX2	-4.43	2.45E-04		3	Upregulated
	NF1P9	-1.62	1.08E-03	13		Not found
	Sep-11	-3.11	1.63E-04	14		Not found
	TCEB3	-4.48	2.42E-04	7		Not found
HuR target genes that show up-regulation upon HuR knockdown in other cell types and are up-regulated in *L. donovani* infection in macrophages	ARID5B	1.69	4.74E-03		12	Upregulated
	ATP13A3	1.51	5.19E-05		30	Downregulated
	BCL10	2.32	1.21E-03	10		Not found
	CD44	1.73	9.78E-04	9	4	Downregulated
	CDH2	4.25	2.60E-04		1	Upregulated
	CLIP1	1.79	3.32E-04	6		Upregulated
	CRKL	3.04	8.47E-03		4	Not found
	DUSP3	2.1	3.51E-05	3		Downregulated
	DYRK1A	7.33	1.17E-04		61	Downregulated
	ECE1	1.75	1.55E-02	2		Upregulated
	EIF4E	2.06	9.79E-04		23	Not found
	FAM110B	2.12	2.18E-02		2	Upregulated
	FUT8	1.58	1.12E-03		16	Downregulated
	GBP1	1.76	1.18E-03	2		Not found
	GCH1	3.44	1.40E-03		6	Upregulated
	HIPK3	1.81	1.92E-02		9	Upregulated
	ITGAV	3.32	8.99E-04	20		Downregulated
	LMAN1	2.29	4.85E-03	10		Upregulated

	MED21	2.74	4.98E-04			Not found
	LPP	2.11	3.09E-02	42	46	Downregulated
	MAP7	2.07	9.05E-03	13		Upregulated
	MAST1	2.82	4.76E-04		2	Downregulated
	MDM2	3.23	4.51E-03		4	Downregulated
	ME1	3.52	1.48E-02	8		Downregulated
	MINPP1	2.13	4.29E-03	3		Upregulated
	MOCS3	1.53	1.17E-03	4		Downregulated
	MPZL1	2.74	1.03E-04	6		Downregulated
	PARD6B	2.7	1.20E-04		3	Not found
	PITPNB	2.42	1.15E-02	12	43	Downregulated
	PMAIP1	2.41	5.99E-06		15	Downregulated
	PPID	6.36	1.44E-04	3		Downregulated
	PPP3R1	3.06	5.16E-03	11		Downregulated
	PRKAR1A	1.92	4.58E-02		18	Not found
	PTPN1	1.53	1.17E-04	3		Downregulated
	RAB31	1.8	5.24E-03	10		Upregulated
	RAB5B	2.79	4.85E-04		5	Downregulated
	RALA	1.59	1.89E-04	8		Downregulated
	RARA	1.76	2.16E-03		2	Upregulated
	RBBP4	1.95	1.52E-02		13	Not found
	RCN2	5.07	1.65E-03	2		Downregulated
	SACS	3.99	4.66E-04	5		Downregulated
	SDC2	1.78	3.20E-02	10		Upregulated
	SMARCD1	2.25	5.58E-03	3		Upregulated
	SNAP23	2.14	3.89E-03		1	Not found
	MATR3	5.6	2.42E-04			Not found
	SSFA2	2.28	2.07E-02	25		Downregulated
	TBC1D5	8.24	2.37E-05	4		Downregulated
	TCF7L2	1.76	9.56E-04		20	Upregulated
	TM4SF1	1.99	1.46E-03	5		Upregulated
	TMED7	1.62	7.44E-03	23		Not found
	TMX1	1.97	1.83E-03	9		Not found
	TP53	1.76	3.09E-02		4	Downregulated
	UAP1	2.21	6.86E-04	3		Upregulated
	UBE2K	2.36	2.97E-02	24		Downregulated
	XIAP	2.27	6.58E-04		24	Downregulated
	YWHAZ	3.18	3.62E-03	10		Downregulated
HuR target genes that show up-regulation upon HuR knockdown in other cell	ETV5	-1.65	3.21E-05		5	Downregulated
	SNCA	-4.43	2.45E-04		17	Downregulated
	MPPED2	-3.45	3.54E-04		1	Upregulated
	COL1A1	-2.57	5.47E-04	1		Upregulated
	PARD6B	-2.28	6.53E-04		3	Not found
	FGF5	-1.88	8.67E-04		4	Downregulated
	PRKAA1	-1.78	9.37E-04	25		Downregulated

types but are down-regulated in *L. donovani* infection in macrophages	ECD	-1.65	1.05E-03	4		Downregulated
	SS18	-1.83	9.44E-04		58	Downregulated
	MKL1	-2	4.01E-03	4		Upregulated
	FSCN1	-2.02	8.38E-03	4		Upregulated
	TRAPPC2	-1.71	1.82E-02			Not found
	ACVR2A	-1.72	3.86E-02		10	Upregulated
HuR target genes that show down-regulation upon HuR knockdown in other cell types but are up-regulated in *L. donovani* infection in macrophages	AMD1	1.81	1.93E-03		17	Downregulated
	ANKRD17	2.08	1.83E-02	27		Downregulated
	ANP32E	1.8	9.01E-03	16		Downregulated
	ATF2	1.52	1.21E-04		41	Downregulated
	BAZ1A	2.07	3.07E-05	4		Not found
	BCL10	2.32	1.21E-03		15	Not found
	CASP2	1.74	5.39E-03		8	Upregulated
	CEP170	2.05	3.80E-05			Not found
	CKS2	5.32	2.70E-02	3		Upregulated
	CLPX	2.08	3.45E-03	2		Upregulated
	CRKL	3.04	8.47E-03	6		Not found
	DDX10	3.03	1.95E-06	5		Downregulated
	DDX21	1.81	4.92E-03	11		Downregulated
	EIF4E	2.06	9.79E-04	9		Not found
	ETS2	2.25	3.19E-03		2	Downregulated
	ETV3	1.6	1.10E-03		9	Downregulated
	EXPH5	2.67	7.36E-04		8	Downregulated
	FECH	2.03	7.32E-03	6		Not found
	FGFR1OP	2.15	1.00E-04	2		Downregulated
	FKBP5	1.66	1.04E-03	3	7	Downregulated
	FXN	2.78	4.86E-04		1	Downregulated
	GEM	1.97	2.35E-03		2	Downregulated
	GNS	1.54	2.52E-02	2		Upregulated
	H1F0	1.74	5.63E-05	4		Downregulated
	HOXD13	6	6.33E-06		1	Downregulated
	IPO5	1.62	2.23E-02	7		Downregulated
	IRF8	3.44	4.69E-03		3	Upregulated
	ISCA1	1.51	6.58E-03	2	7	Downregulated
	ITPR3	2.77	4.90E-04	1		Upregulated
	KLHL20	1.53	1.29E-05		15	Upregulated
	KYNU	1.59	4.86E-04	13		Upregulated
	LIFR	1.81	9.17E-04		3	Upregulated
	SOD2	4.49	1.48E-03	10		Not found
	RRN3	5.12	1.98E-04	5		Not found
	MAN1A1	2.03	6.67E-03		14	Upregulated
	MAN1A2	5.34	1.86E-04	9		Downregulated
	MAX	1.57	5.31E-03	6	5	Upregulated
	MBNL2	2.16	1.07E-02		1	Downregulated

	MET	2.58	3.26E-03	5		Downregulated
	MYO1B	2.16	7.64E-04	2		Downregulated
	N4BP2L2	2.57	5.48E-04		94	Downregulated
	NUDT21	1.54	1.79E-05	12		Upregulated
	NUP153	1.8	2.62E-04	5		Not found
	OXCT1	2.13	7.20E-04	10		Downregulated
	PFN2	3.69	4.27E-02	6	6	Upregulated
	PLOD2	2.5	1.58E-03	16	4	Upregulated
	POLR2D	5.63	1.66E-06		1	Upregulated
	PPFIA1	4.77	2.38E-04	2		Not found
	PPP2CA	3.18	3.99E-04	8		Downregulated
	PRKAR1A	1.92	4.58E-02	11		Not found
	PSIP1	1.5	4.25E-04	8		Downregulated
	PTPN12	1.67	2.56E-04	4		Downregulated
	RBBP4	1.95	1.52E-02	6		Not found
	RBMS3	2.04	7.71E-04		3	Downregulated
	RDX	2.28	4.21E-02	12		Downregulated
	RIF1	1.91	1.67E-03	8		Downregulated
	RSBN1	2.36	6.26E-03		9	Downregulated
	SGPL1	4.58	1.41E-02	4		Downregulated
	SIKE1	2.64	3.66E-03		7	Upregulated
	SLC7A11	3.43	3.59E-03	55		Downregulated
	SNAP23	2.14	3.89E-03	5		Not found
	SNHG4	5.6	2.42E-04	17		Not found
	SPC25	1.55	1.16E-03		7	Downregulated
	SPTBN1	3.7	3.20E-04	12		Downregulated
	STAG2	3.19	3.72E-03	9		Downregulated
	STX16	1.91	8.50E-04	5		Downregulated
	TCEB3	2.48	3.30E-03	7		Not found
	TFDP2	2.5	7.21E-06		19	Not found
	TFPI	1.9	1.06E-04		12	Downregulated
	TFRC	4.01	1.86E-02	10		Downregulated
	TLN2	1.96	1.03E-03	2		Upregulated
	TMX1	1.97	1.83E-03		5	Not found
	TROVE2	1.66	1.04E-03	27	29	Downregulated
	TSPYL1	1.73	3.99E-03	6	5	Upregulated
	UBE2D1	5.08	6.68E-03		21	Upregulated
	UBE2N	2.39	6.09E-04		20	Downregulated
	USP13	4.66	2.28E-04	2		Upregulated
	RAB6A	5.3	1.65E-02		7	Not found
	YME1L1	1.55	3.66E-02	7		Downregulated
	ZBTB1	1.88	8.64E-04		9	Downregulated

Note: Herein, upregulated is indicative of fold change > 0 and downregulated is indicative of fold change < 0

Appendix Table S2: List of experiments and P values

Figure No.	Description	P-Value	Method used
**Figure 1**			
1B	0hr-3hr	0.0006	Paired t test
	0hr-6hr	0.0038	Paired t test
	0hr-24hr	0.2992	Paired t test
1E	0hr-4hr	0.0181	Paired t test
	0hr-24hr	0.902	Paired t test
	4hr-24hr	0.0071	Unpaired t test
1H	siCon 24hr-siPP2A 24hr	0.0169	Unpaired t test
	siCon 24hr-siMKP1 24hr	0.3059	Unpaired t test
	siCon 24hr-siMKP3 24hr	0.0867	Unpaired t test
	siCon 24hr-siSHP1/2 24hr	0.2625	Unpaired t test
1K	siCon-siPP2A (let7a)	0.001	Paired t test
	siCon-siPP2A (miR155)	0.0002	Paired t test
	siCon-siPP2A (miR146a)	0.0039	Paired t test
1L	siCon-siPP2A (IL-1β)	0.0198	Paired t test
**Figure 2**			
2C	24hrLPS-24hrLPS with OA	0.0009	Unpaired t test
2F	14hr DOX without OA-24hr DOX without OA	0.0216	Paired t test
	14hr DOX with OA-24hr DOX with OA	0.1241	Paired t test
	24hr DOX without OA-24hr DOX with OA	0.0056	Unpaired t test
2G	24hr DOX without OA-24hr DOX with OA	0.1365	Unpaired t test
2J	siCon-siPTPA (let7a)	0.0187	Paired t test
	siCon-siPTPA (miR146a)	0.004	Paired t test
**Figure 3**			
3C Upper panel	0hr-24hr (IL-10)	0.0367	Paired t test
	0hr-24hr (TNF-a)	0.0463	Paired t test
	0hr-24hr (IL-6)	0.0374	Paired t test
3C lower panel	0hr-24hr (IL-10)	0.04658	Paired t test

	0hr-24hr (TNF-a)	0.038	Paired t test
	0hr-24hr (IL-6)	0.04215	Paired t test
**Figure No.**	**Description**	**P-Value**	**Method used**
3E	0hr-4hr Ld	0.0344	Paired t test
3G	- Ld vs + Ld	0.03985	Unpaired t test
3I	siCon 0hr-siCon 4hr	0.0202	Paired t test
	siPP2A 0hr-siPP2A 4hr	0.076	Paired t test
	siCon 4hr-siPP2A 4hr	0.0179	Unpaired t test
**Figure 4**			
4A	siCon 24hr- siPP2A 24hr (IL-10)/RAW264.7	0.0222	Unpaired t test
	siCon 24hr- siPP2A 24hr (IL-10)/PEC	0.0107	Unpaired t test
	siCon 24hr- siPP2A 24hr (IL-6)/RAW264.7	0.0048	Unpaired t test
	siCon 24hr- siPP2A 24hr (IL-6)/PEC	0.0017	Unpaired t test
	siCon 24hr- siPP2A 24hr (IL-6)/RAW264.7 [Elisa]	0.0008	Unpaired t test
4C left panel	siCon-siPP2A	< 0.0001	Unpaired t test
4C Right panel	siCon-siPP2A	0.0149	Paired t test
4D	siCon 24hr-siPP2A 24hr (WT)	0.03365	Unpaired t test
	siCon 24hr-siPP2A 24hr (Y529F)	0.1196	Unpaired t test
4F left panel	WT-Y529F (TNF-a)	0.4137	Paired t test
	WT-Y529F (IL-1β)	0.0151	Paired t test
	WT-Y529F (IL-10)	0.0199	Paired t test
4F Right panel	WT-Y529F	< 0.0001	Unpaired t test
4G	2hours without OA-2hours with OA	0.2492	Unpaired t test
	24hours without OA-24hours with OA	0.0004	Unpaired t test
**Figure 5**			
5B	-LPS+LPS (Let-7a)	0.0211	Unpaired t test
5C	-LPS +LPS (let-7a)/(miR-155)	0.0264/ 0.0393	Paired t test
5D	- GW4869, +GW4869 (let-7a /miR-155)	0.0023 /0.00053	Paired t test
5E	- GW4869, +GW4869 (TNF-α /IL-6)	0.0009/ 0.0021	Paired t test
5F	- siCon, siHuR(TNF-α)/IL-6/IL-1β/miR-155)	0.0329/0.0381/0.0193/ 0.0221	Paired t test

5G	siCon-siHuR	0.0009	Paired t test
**Figure No.**	**Description**	**P-Value**	**Method used**
5I left panel	pCIneo-HA HUR(without LPS)	0.0592	Paired t test
	pCIneo-HA HUR(with LPS)	0.0149	Unpaired t test
5I right panel	pCIneo-HA HUR(without LPS)	0.0086	Unpaired t test
	pCIneo-HA HUR(with LPS)	0.0111	Unpaired t test
5L lower panel	without MG132-with MG132	0.015	Paired t test
**Figure 6**			
6C	pCIneo-HA HUR (PP2A A subunit)	0.0018	Paired t test
	pCIneo-HA HUR (PP2A B subunit)	0.0021	Paired t test
	pCIneo-HA HUR (PP2A Cα subunit)	0.0171	Paired t test
**Figure 7**			
7B	untransfected-HA HUR transfected	< 0.0001	Unpaired t test
7C	pCIneo-HA HUR 24hr Ld(TNF-a)	0.019	Paired t test
	pCIneo-HA HUR 24hr Ld(IL-1β)	0.0214	Paired t test
	pCIneo-HA HUR 24hr Ld(IL-10)	0.0024	Paired t test
7D	uninfected- 10days infection	0.0003	Unpaired t test
	uninfected- 35days infection	< 0.0001	Unpaired t test
7G	pCI neo-HAHUR (Ld count) dot plot	0.0026	Unpaired t test
	pCI neo-HAHUR (Ld count) histogram	0.0002	Paired t test
7H	pCI neo-HAHUR (TNF-a) dot plot	0.0069	Unpaired t test
	pCI neo-HAHUR (TNF-a) histogram	0.0249	Paired t test
7I	pCI neo-HAHUR (IL-10) dot plot	0.0119	Unpaired t test
	pCI neo-HAHUR (IL-10) histogram	0.0258	Paired t test

7K	pCI neo-HAHUR (TNF-a) histogram Spleen	0.7299	Paired t test
**Figure No.**	**Description**	**P-Value**	**Method used**
**Figure 8**			
8A	control-Lds (IL-10)	0.0006	Paired t test
	control-Ldr (IL-10)	0.035	Paired t test
	Lds-Ldr (IL-10)	0.0277	Unpaired t test
8B	control-Lds (TNF-a)	0.0267	Paired t test
	control-Ldr (TNF-a)	0.024	Paired t test
8C	control-Lds	0.0105	Paired t test
	control-Ldr	0.0115	Paired t test
	Lds-Ldr	0.0061	Unpaired t test
8F	pciNeo without OA-pciNeo with OA (IL-1β)	0.0004	Paired t test
	HA HUR without OA-HA HUR with OA (IL-1β)	0.0088	Paired t test
	pciNeo without OA-pciNeo with OA (TNF-a)	0.703	Paired t test
	HA HUR without OA-HA HUR with OA (TNF-a)	0.0177	Paired t test
8H	pciNeo without OA-HA HUR with OA (TNF-a)	0.026	Paired t test
	HA HUR without OA-HA HUR with OA (TNF-a)	0.012	Unpaired t test
	pciNeo without OA-HA HUR with OA (IL-1β)	0.0056	Paired t test
	HA HUR without OA-HA HUR with OA (IL-1β)	0.0018	Unpaired t test
	pciNeo without OA-HA HUR with OA (Ld count)	< 0.0001	Paired t test
	HA HUR without OA-HA HUR with OA (Ld count)	0.0001	Unpaired t test
**Figure EV1**			
EV1E	0h/24h LPS PP2AA	0.0493	Paired t test
	0h/24h LPS PP2AB	0.0015	Paired t test
	0h/24h LPS PP2ACα	0.0003	Paired t test
**Figure EV2**			
EV2B	control-Ld 24hr	0.0011	Paired t test
	Ld 24hr-Ld 24hr heat killed	0.0003	Unpaired t test

EV2F	control-LPS 4hr	0.0011	Paired t test
	control-LPS 4hr+anti TLR4	0.0036	Paired t test
	LPS 4hr-LPS 4hr+anti TLR4	< 0.0001	Unpaired t test
**Figure No.**	**Description**	**P-Value**	**Method used**
EV2G	control-LPS 4hr	0.0059	Paired t test
	control-LPS 4hr+anti TLR4	0.01	Paired t test
	LPS 4hr-LPS 4hr+anti TLR4	0.0137	Unpaired t test
EV2H	control-1U LPG	0.1412	Paired t test
	control-10U LPG	0.0279	Paired t test
	10U LPG- 10U LPG+anti TLR4	0.0163	Unpaired t test

Appendix Table S3 List of Primers used for mRNA detection

Target	5'Forward Primer3'	5'Reverse Primer3'
TNF-α	GTCTCAGCCTCTTCTCATTCC	TCCACTTGGTGGTTTGCTA
IL-1β	GACCTTCCAGGATGAGGACA	CCTTGTACAAAGCTCATGGAG
IL-6	AGGATACCACTCCCAACAGA	GTACTCCAGAAGACCAGAGGA
IL-10	TGCTAACCGACTCCTTAATGC	ATCACTCTTCACCTGCTCCAC
RL	CCAAGCAAGATCATGC	GCTCTTGATGTACTTACCC
PP2A-α	GGCCTGTGGTCTCTTCTCAG	CTCGTCAGAGGCCAGGTTAG
PP2A-β	AGCTGGCGTACTGTGTTGTG	GGAAATGAGAGCTGGAGACG
PP2A-C_{α}	AGAGCGCATCACCATACTCC	CCAAGGCAGTGAGAGGAAGA
18SrRNA	TGACTCTAGATAACCTCGGG	GACTCATTCCAATTACAGGG
Parasite specific primer	CCTATTTTACACCAACCCCCAGT (JW11)	GGGTAGGGGCGTTCTGCGAAA (JW12)

Appendix Table S4 List of antibodies used

Antibody names	Raised in	Dilution	Source
4G10	Mouse	1:1000	Millipore
AGO2 (EIF2C2)	Mouse	1:1000	Abnova
Alix	Mouse	1:200	Santa cruz
β-Actin	Mouse Monoclonal (HRP conjugated)	1:10000	Sigma Aldrich
HA	Rat monoclonal	1:1000	Roche
HRS	Rabbit	1:1000	Bethyl
HuR	Mouse	1:1000	Santa cruz
MKP1	Rabbit	1:1000	Kind gift from Dr. Pijush Kanti Das
MKP3	Rabbit	1:1000	Kind gift from Dr. Pijush Kanti Das
MSK1/2	Rabbit	1:1000	Cell signalling
PP2A	Rabbit	1:1000	Santacruz
PTPA	Mouse	1:1000	Millipore
p38	Rabbit	1:1000	Cell signalling
p-P38	Rabbit	1:1000	Cell signalling
p-Msk1/2	Rabbit	1:1000	Cell signalling
SHP1/2	Rabbit	1:1000	Kind gift from Dr. Pijush Kanti Das
ERK	Rabbit	1:1000	Cell signalling
p-ERK	Rabbit	1:1000	Cell signalling
Dicer1	Rabbit	1:5000	Bethyl
MDR1	Rabbit	1:500	Novus Biologicals

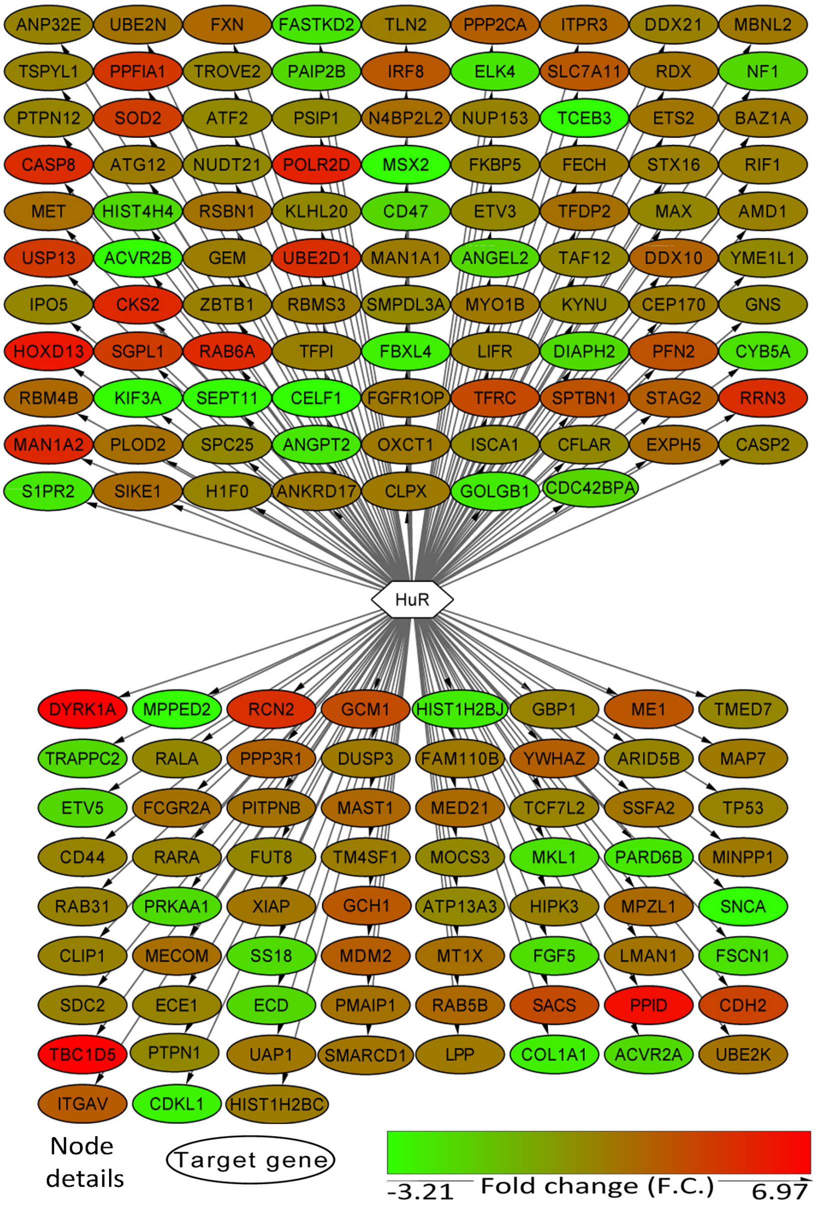

Appendix Figure S1 Status of HuR target genes in *L. donovani* infected macrophages. HuR regulated genes that may be targeted in Ld infected cells based on their differential expression upon *L. donovani* infection in macrophages is outlined here. Note: The node colour is indicative of fold change (F.C.) in gene expression of respective HuR target genes in *L. donovani* infected macrophages. The complete set of differentially expressed probable HuR regulated genes have been listed in Appendix Table S1.
