# Supplementary material for: MicroRNA exporter HuR clears the internalized pathogens by promoting pro‐inflammatory response in infected macrophages
Source: EMBO Mol Med. 2020 Feb 7;12(3):e11011. doi: 10.15252/emmm.201911011 (PMC7059013; doi:10.15252/emmm.201911011)
Supplement: Supplementary file 2 — Expanded View Figures PDF [file EMMM-12-e11011-s002.pdf]

Expanded View Figures

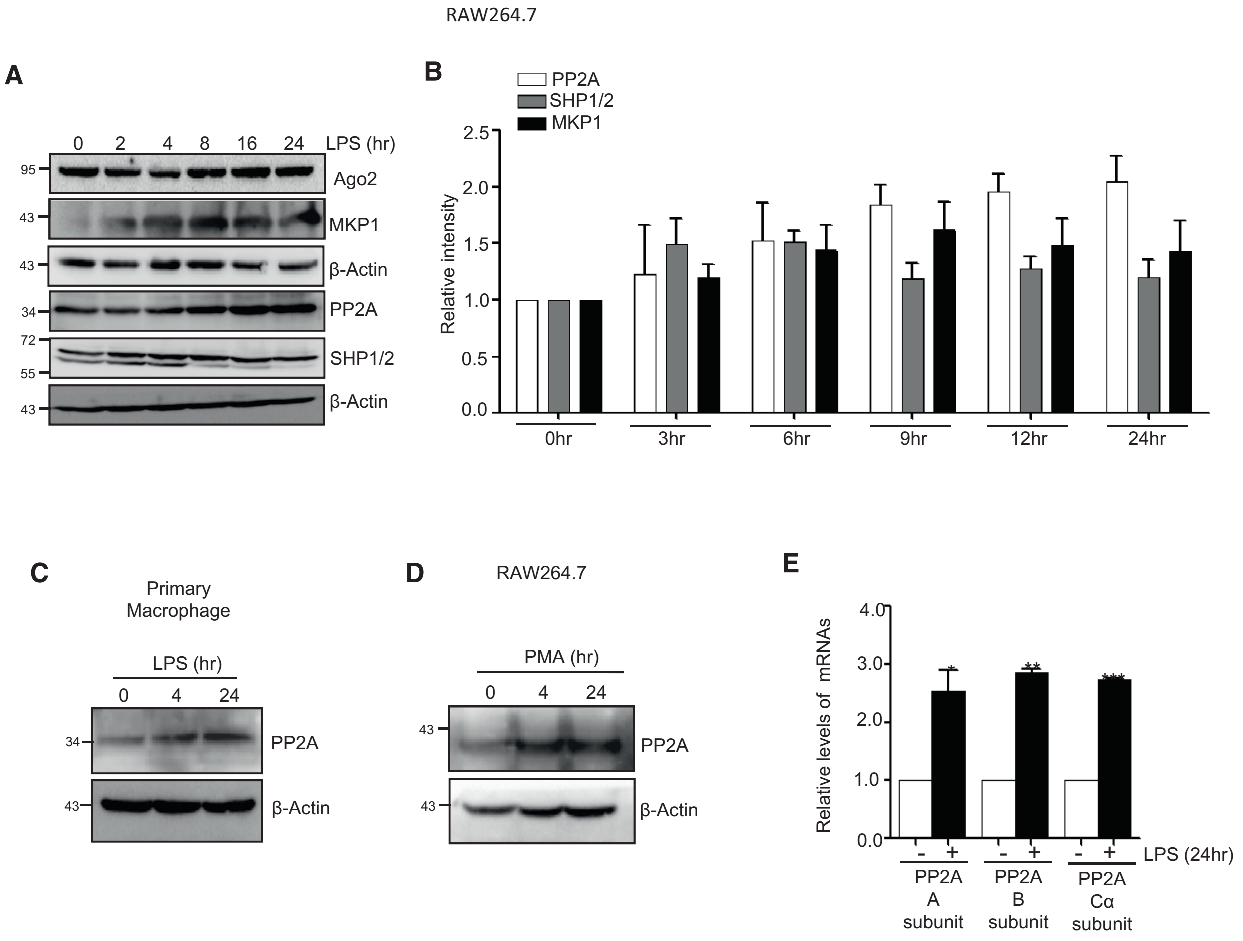

Figure EV1. Expression of PP2A in macrophage cells.

A Levels of Ago2 and different phosphatases during the course of LPS stimulation were determined in Western blots done with total cell lysate. β-Actin was used as loading control.

B Relative intensity of PP2A, SHP1/2 and MKP1 was quantified using ImageJ. Every lane was normalized with respective β-Actin band. Quantification from triplicate experimentations.

C Expression of PP2A observed in mouse peritoneal exudate cells (PEC) primary cells treated with LPS against time.

D Expression of PP2A determined by Western blot analysis in RAW 264.7 cells treated with phorbol 12-myristate 13-acetate (PMA).

E Relative levels of three transcript variants of PP2A in LPS-treated cells. Relative levels in untreated cells were considered as unit.

Data information: For Western blot, β-Actin was used as loading control while for RNA estimation 18S rRNA was used as control. In all experimental data, ns: non-significant and *, ** and *** represent *P*-value of < 0.05, < 0.01 and < 0.001, respectively, quantified by using Student's *t*-test. Data in panels (B and E) are from experiments done in triplicates (mean ± s.e.m., $n = 3$). Exact *P*-values against each experimental set are presented in the Appendix Table S2. Positions of molecular weight markers are marked and shown in the Western blots used in different panels.
Source data are available online for this figure.

© 2020 The Authors

Figure EV2. Effect of phagocytosis and pathogen-derived LPG on host cell PP2A expression.

A, B RAW 264.7 cells were treated with living and heat killed parasites (attenuation done at 100°C water bath for 10 min) for given time points, and the Ago2 and PP2A levels were estimated by Western blots. β-Actin was used as a loading control (A). Subsequently, PP2A-Cα mRNA level was also estimated for 24-h time point using qRT–PCR. 18S rRNA was used as an endogenous control (mean ± s.e.m., *n* = 3) (B).

C–E Latex bead phagocytosis in RAW 264.7 cells. A schematic representation of latex bead phagocytosis assay (C). RAW 264.7 cells were treated with latex beads (fluorescent red, Sigma-Aldrich) for different time points. In brief, latex beads were diluted in complete RPMI medium and added to cell in the same at 10:1 (bead to cell ratio) for 2, 4, 8, 16 and 24 h. Red fluorescent-labelled beads were visualized using confocal microscope (D). DIC images with red and blue (DAPI) channels are shown as superimposed images. PP2A-Cα mRNA and protein levels were estimated using RT–PCR and Western blot analysis, respectively (average ± SD, *n* = 2) (E). β-Actin and 18S rRNA were used as controls in Western blot and qRT-PCR respectively.

F, G TLR4 mediated PP2A upregulation during LPS stimulation. RAW 264.7 cells untreated, 4 h of LPS (1 ng/ml) treatment and 4 h of LPS (1 ng/ml) treatment in presence of anti-TLR4 antibody (10 μg/ml) were examined for PP2A-Cα (F) and TNF-α (G) expression. Cells treated with anti-TLR4 antibody were pre-treated for 2 h prior to LPS stimulation (mean ± s.e.m., *n* = 4).

H–J Effect of *Ld*-derived LPG on RAW 264.7 cells. Cells untreated or pre-treated with anti-TLR4 antibody (10 μg/ml) for 2 h with anti-TLR4 antibody were exposed to increasing concentration of LPG for another 2 h and were examined for PP2A-Cα mRNA (H) (mean ± s.e.m., *n* = 4) and PP2A protein expression (mean ± s.e.m., *n* = 2) (J). TNF-α mRNA level was quantified during similar condition (mean ± s.e.m., *n* = 4) (I). Band intensities were normalized against β-Actin bands, and the relative values were plotted (mean ± s.e.m., *n* = 2).

Data information: For Western blot, β-actin was used as loading control while for RNA estimation 18S rRNA was used as control. 1U of LPG is equivalent to amount that induces 1.5-fold increase of TNF-α production in treated naive RAW 264.7 cells. In all experimental data, ns: non-significant and *, ** and *** represent *P*-value of < 0.05, < 0.01 and < 0.001, respectively, calculated using Student's *t*-test. For statistical analysis, all experiments were done three times. Exact *P*-values against each experimental set are presented in the Appendix Table S2. Positions of molecular weight markers are marked and shown in the Western blots used in different panels. Scale bar for panel (D) is 20 μm.
Source data are available online for this figure.

© 2020 The Authors

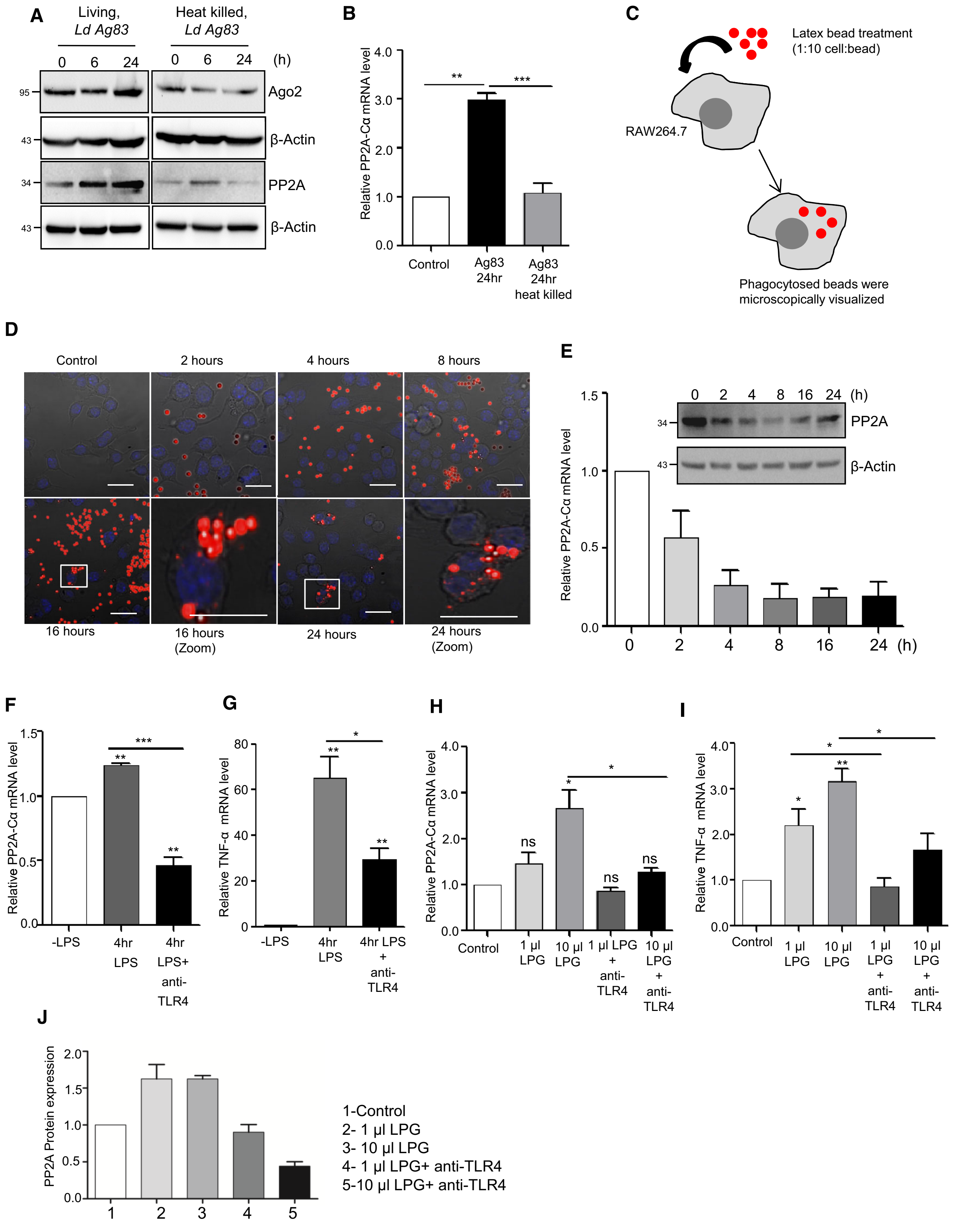
A
Living, Ld Ag83
Heat killed, Ld Ag83
0 6 24 0 6 24 (h)
95
Ago2
43
β-Actin
34
PP2A
43
β-Actin
B
Relative PP2A-Cα mRNA level
4.0
3.0
2.0
1.0
0.0
**
***
Control
Ag83 24hr
Ag83 24hr heat killed
C
Latex bead treatment (1:10 cell:bead)
RAW264.7
Phagocytosed beads were microscopically visualized
D
Control
2 hours
4 hours
8 hours
16 hours
16 hours (Zoom)
24 hours
24 hours (Zoom)
E
Relative PP2A-Cα mRNA level
1.5
1.0
0.5
0.0
0 2 4 8 16 24 (h)
34
PP2A
43
β-Actin
F
Relative PP2A-Cα mRNA level
1.5
1.0
0.5
0.0
***
**
**
-LPS
4hr LPS
4hr LPS+ anti-TLR4
G
Relative TNF-α mRNA level
80
60
40
20
0
*
**
**
-LPS
4hr LPS
4hr LPS + anti-TLR4
H
Relative PP2A-Cα mRNA level
4.0
3.0
2.0
1.0
0.0
ns
*
ns
ns
*
Control
1 µl LPG
10 µl LPG
1 µl LPG + anti-TLR4
10 µl LPG + anti-TLR4
I
Relative TNF-α mRNA level
4.0
3.0
2.0
1.0
0.0
*
*
*
**
Control
1 µl LPG
10 µl LPG
1 µl LPG + anti-TLR4
10 µl LPG + anti-TLR4
J
PP2A Protein expression
2.0
1.5
1.0
0.5
0.0
1 2 3 4 5
1-Control
2- 1 µl LPG
3- 10 µl LPG
4- 1 µl LPG+ anti-TLR4
5-10 µl LPG+ anti-TLR4

Figure EV2.

Figure EV3. Cleavage of HuR by *Leishmania* membrane protease GP63.

A Cleavage of HuR by *Ld* membrane-derived soluble *Leishmania* antigen (SLA). The HuR cleavage is prevented by ortho-phenanthroline (OPT). Protein equivalent amount of cell extract (100 µg) from RAW 264.7 cells were incubated with SLA (1 µg) in presence and absence of OPT for 30 min at 37°C, and level of HuR in treated cell lysate was determined by Western blot analysis. β-Actin was used as loading control.

B–E Cleavage of HuR by purified Gp63. The silver-stained gel containing different fractions obtained during the dual steps purification of the GP63 from SLA derived from *Ld* membranes. Arrow mark the GP63 band (B). Increasing concentration of purified GP63 was incubated with fixed quantity of RAW 264.7 cell lysate and HuR cleavage reaction was done and HuR levels in each reaction were detected by Western blot (C). GP63 treatment cleaves HuR but it has no effect on β-Actin or Alix present in the RAW 264.7 lysate. This denotes the specificity of this cleavage reaction (D). The HuR cleavage by purified GP63 can be blocked by OPT (E).

F–I Schematic representation of GP63 entrapped liposome formation has been depicted (F). Purified GP63 was entrapped in specific liposome, and the liposome size and concentration were estimated using nanoparticle tracker. Size of the empty liposome was around 175.7 nm, and GP63 entrapped liposome was around 191.8 nm. Concentration of empty liposome was estimated to be around 6×10^8 particles/ml, and GP63 entrapped liposome was in the range of 6×10^7 particles/ml (G). RAW 264.7 cells were treated with 1 µg of GP63 entrapped liposome and incubated for 6 and 16 h, after which cells were harvested to check for HuR levels. Visible reduction in HuR levels was observed along with unchanged Ago2 levels, suggesting a specific cleavage action on HuR. Empty liposome treatment was used as a control (H). GP63-containing liposomes were used to cleave HuR in RAW 264.7 cells. C1 and C2 are control set with empty liposome treated, and gp1, gp2 and gp3 are GP63 liposome-treated cells. HuR and Dicer1 levels were checked in cell lysates. Dicer1 has been previously reported to be cleaved by GP63 and hence was used as positive control. β-Actin was used as loading as well as negative control (I). Positions of molecular weight markers are marked and shown in the Western blots used in different panels.

Source data are available online for this figure.

© 2020 The Authors

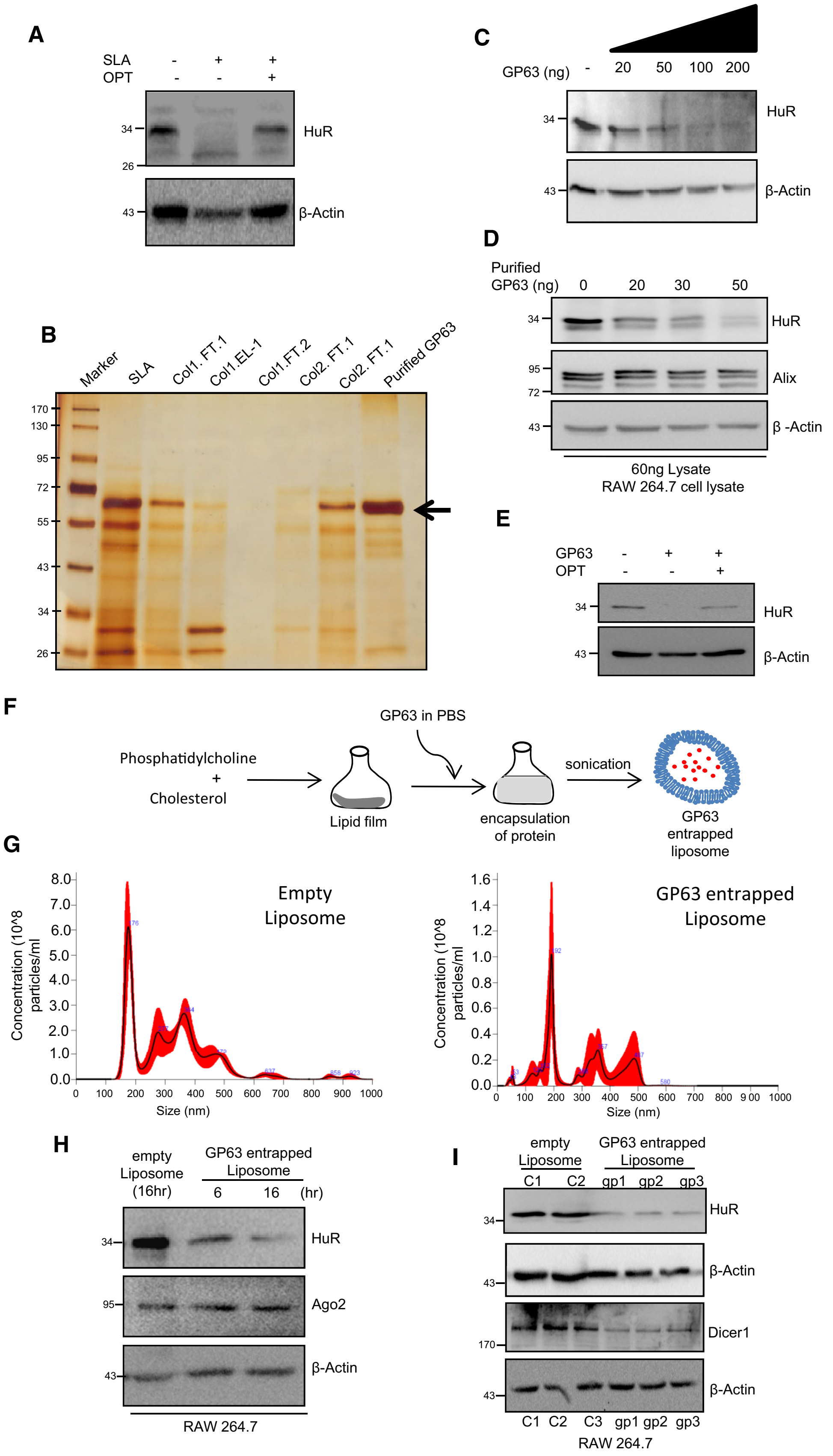
A
SLA - + +
OPT - - +
HuR
β-Actin
B
Marker SLA Col1. FT.1 Col1.EL-1 Col1.FT.2 Col2. FT.1 Col2. FT.1 Purified GP63
C
GP63 (ng) - 20 50 100 200
HuR
β-Actin
D
Purified GP63 (ng) 0 20 30 50
HuR
Alix
β -Actin
60ng Lysate
RAW 264.7 cell lysate
E
GP63 - + +
OPT - - +
HuR
β-Actin
F
Phosphatidylcholine + Cholesterol
Lipid film
GP63 in PBS
encapsulation of protein
sonication
GP63 entrapped liposome
G
Empty Liposome
GP63 entrapped Liposome
Concentration (10^8 particles/ml
Size (nm)
H
empty Liposome (16hr)
GP63 entrapped Liposome
6 16 (hr)
HuR
Ago2
β-Actin
RAW 264.7
I
empty Liposome
GP63 entrapped Liposome
C1 C2 gp1 gp2 gp3
HuR
β-Actin
Dicer1
β-Actin
C1 C2 C3 gp1 gp2 gp3
RAW 264.7

Figure EV3.

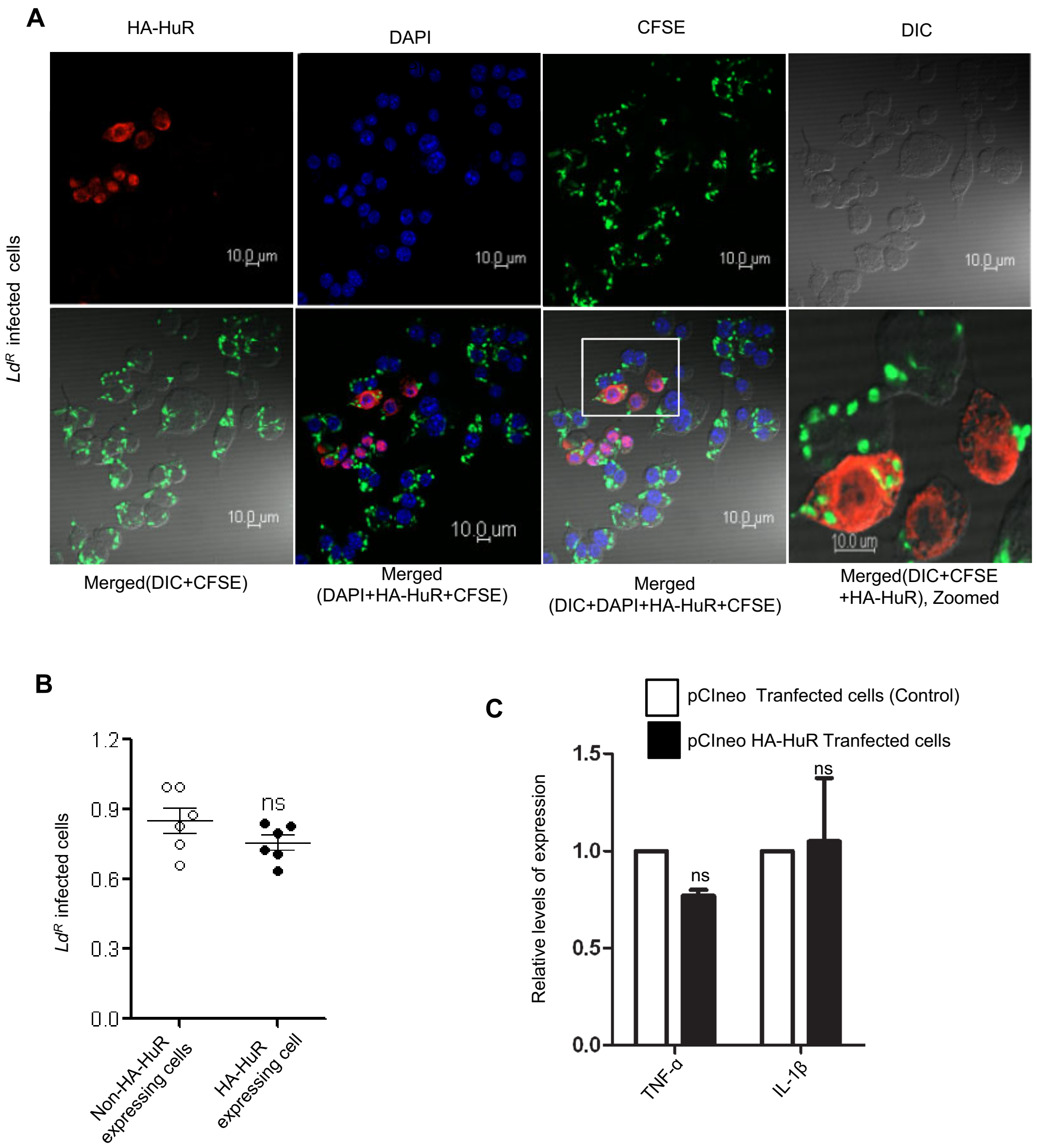

Figure EV4. Overexpression of HuR alone failed to prevent the anti-inflammatory response caused by the drug-resistant parasite Ld^R.

A, B Internalization of *Ld* (Ld^R and Ld^S) in RAW 264.7 cells. Cells were pre-transfected with HA-HuR expression plasmid and infected with CFSE labelled Ld^R parasite (with 1:10 host:parasite ratio) for 24 h, and parasite internalization was imaged using confocal microscope (A). HA-HuR-transfected cells were stained for HA (red), and parasites were labelled with CFSE dye (green) prior to infection. DAPI was used to label nuclei. Number of infected cells was counted from control and HA-HuR-expressing cells (B). Values are mean ± s.e.m., $n = 6$.

C Expression of cytokines in cells infected with Ld^R strain of *Ld*. Cytokine mRNA levels like TNF-α and IL-1β were quantified by real-time PCR from pCIneo or HA-HuR-transfected RAW 264.7 cells after 24-h infection with Ld^R parasites mean ± s.e.m., $n = 3$.

Data information: For statistical analysis, all experiments are done three times and the *P*-values were calculated using Student's *t*-test. Exact *P*-values against each experimental set are presented in the Appendix Table S2.

 © 2020 The Authors
