# Supplementary material for: MicroRNA exporter HuR clears the internalized pathogens by promoting pro‐inflammatory response in infected macrophages
Source: EMBO Mol Med. 2020 Feb 7;12(3):e11011. doi: 10.15252/emmm.201911011 (PMC7059013; doi:10.15252/emmm.201911011)
Supplement: Supplementary file 3 — Source Data for Expanded View [file EMMM-12-e11011-s011.zip › Source_Data_for_EV_Figures/Source_Data_for_FigEV1.pdf]

Figure EV1 Goswami *et al*. Source Data File

Fig EV1 A

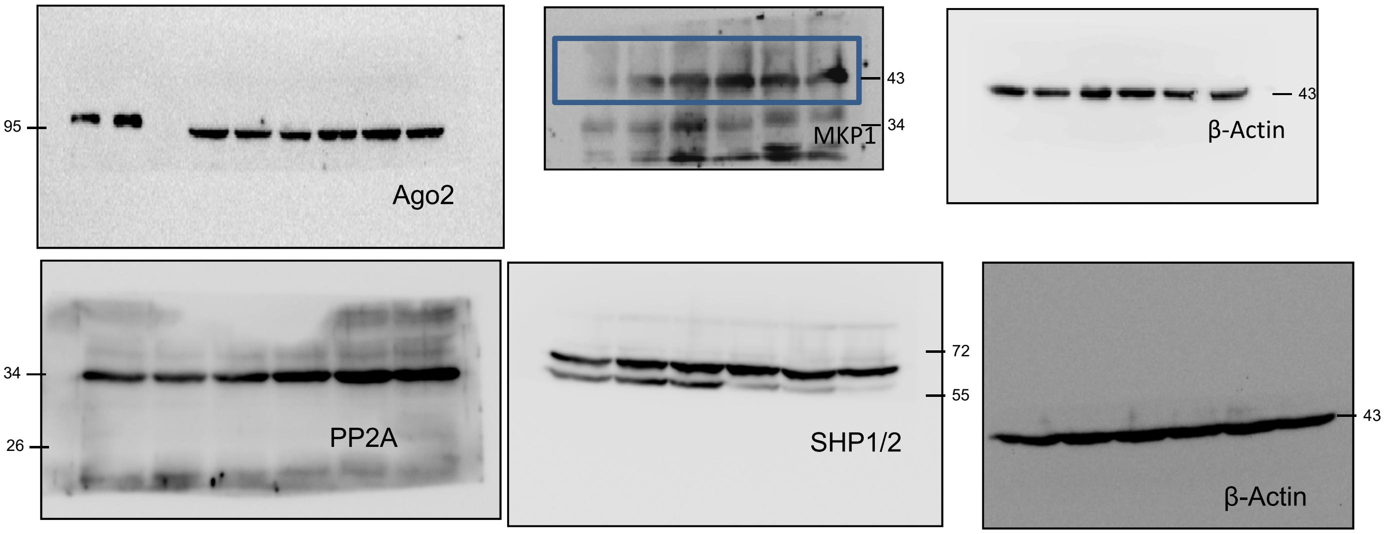

Fig EV1 C

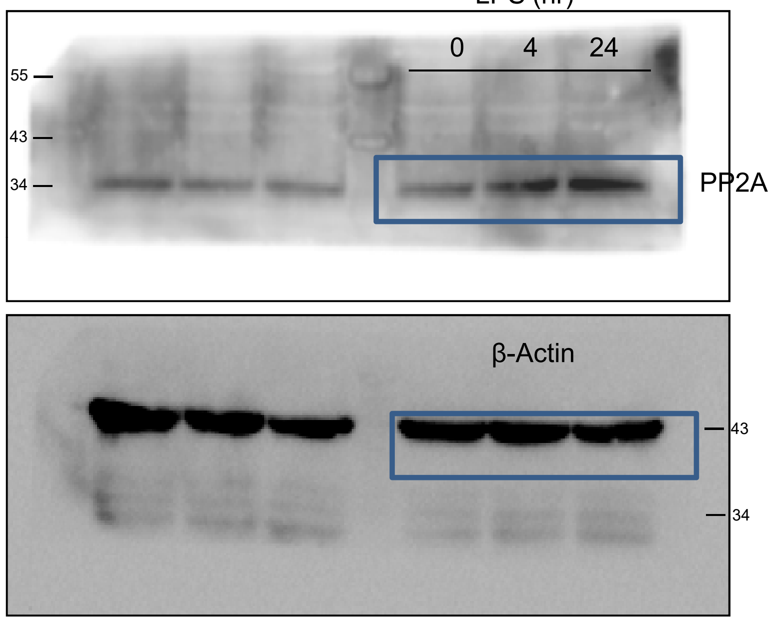

Fig EV1 D

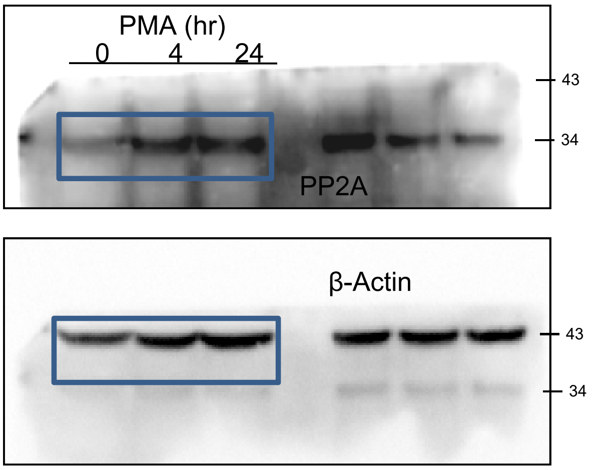

EV1 B

- oa liver bhu tnfa
- oa liver bhu il1b
- PP2A LPG
- tnfa LPG
- oa liver bhu ld count
- parasite count oa 2 24 hr
- **lps phosphatases**
- Data 106
- wt yf ld internalized
- hahur ag83 internalized
- pci HAHUR_LPS_TNFa
- pp2a phosphatase assay

Table format: Grouped	A			B			C	
	PP2A			SHP1/2			MKP1	
	A:Y1	A:Y2	A:Y3	B:Y1	B:Y2	B:Y3	C:Y1	C:Y2
1 0hr	1.000000	1.000000	1.000000	1.000000	1.000000	1.000000	1.000000	1.000000
2 3hr	0.523363	1.140093	2.025304	1.258222	1.947183	1.280662	1.311033	1.087345
3 6hr	0.962245	1.489333	2.123521	1.369928	1.700516	1.477248	1.657718	1.219100
4 9hr	1.722322	1.589494	2.194873	1.175484	0.952099	1.425734	1.870357	1.362968
5 12hr	2.161132	1.659536	2.063548	1.070018	1.452879	1.297240	1.725314	1.234044
6 24hr	2.380395	1.610896	2.154613	0.920151	1.443819	1.235000	1.700266	1.165514

EV1 E

- pci HAHUR_LPS_TNFa
- pp2a phosphatase assay
- Data 111
- Data 112
- **PP2A subunits +- LPS**
- Data 114
- Data 115

	0hr	24hr	0hr	24hr	0hr	24hr
	Y	Y	Y	Y	Y	Y
1	1	2.136986	1	2.945943	1	2.678226
2	1	2.243722	1	2.906848	1	2.787640
3	1	3.255540	1	2.715404	1	2.736702
