# Supplementary material for: MicroRNA exporter HuR clears the internalized pathogens by promoting pro‐inflammatory response in infected macrophages
Source: EMBO Mol Med. 2020 Feb 7;12(3):e11011. doi: 10.15252/emmm.201911011 (PMC7059013; doi:10.15252/emmm.201911011)
Supplement: Supplementary file 3 — Source Data for Expanded View [file EMMM-12-e11011-s011.zip › Source_Data_for_EV_Figures/Source_Data_for_FigEV2.pdf]

Figure EV2 Goswami *et al*. Source Data File

Fig EV 2 A

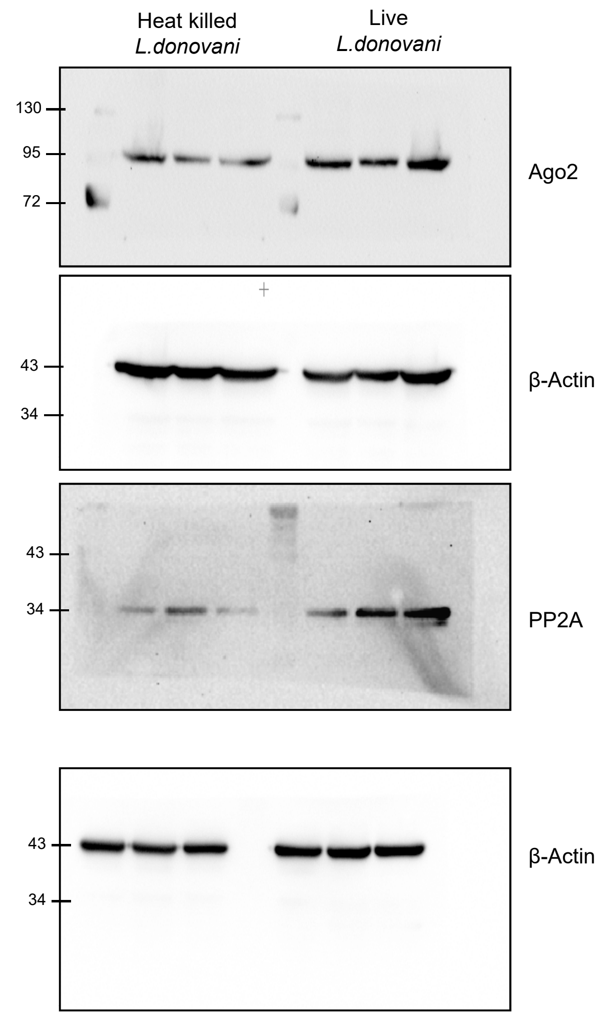

Fig EV 2 E

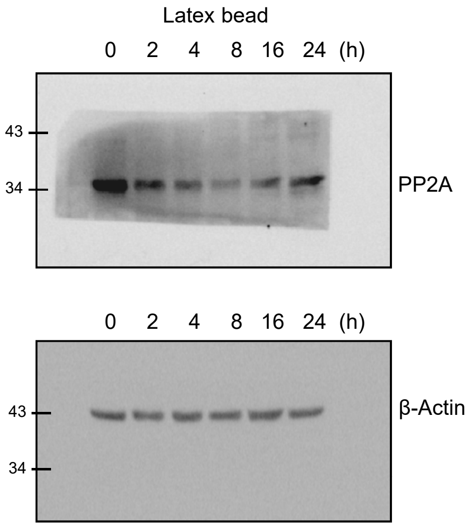

FIG EV 2B

Data 72
LPS mathematical
Data 74
Data 75
ld mathematical
sicon sipp2a_ld
pp2a c ld lm infect
pp2a subunit +- LPS
Ld mathematical
Data 81

	A	B	C
	control	ag83_24hr	24hr_heat killed
	Y	Y	Y
1	1.000000	2.777485	1.310813
2	1.000000	3.154166	1.515244
3	1.000000	3.319708	0.771606
4	1.000000	2.655089	0.715210

FIG EV 2E lower panel

Family
Data Tables
PP2A C
Info
Project info 1
Results
Graphs
PP2A C

	A	B	C	D	E	F
	0 hr	2 hr	4 hr	8 hr	16 hr	24 hr
	Y	Y	Y	Y	Y	Y
1	1.000000	0.328356	0.155322	0.164558	0.266093	0.362654
2	1.000000	0.329877	0.554785	0.440333	0.292532	0.345478
3	1.000000	1.075494	0.223240	0.079476	0.139984	0.064257
4	1.000000	0.544624	0.123850	0.041045	0.051952	0.020381

FIG EV 2F

pp2a c subunit heat killed
l major cytokine tnf il10
heat killed tnfa level
heat killed il 10 level
Data 91
anti tlr 4 PP2A
anti tlr4 TNFa
phospho ago2 lps
pec ago2 associated let7a

	A	B	C
	-LPS	4hr LPS	4hr LPS+α-TLR4
	Y	Y	Y
1	1.000000	1.185504	0.345080
2	1.000000	1.259340	0.638017
3	1.000000	1.271325	0.390934
4	1.000000	1.233423	0.476319

FIG EV 2G

pp2a c subunit heat killed
l major cytokine tnf il10
heat killed tnfa level
heat killed il 10 level
Data 91
anti tlr 4 PP2A
anti tlr4 TNFa
phospho ago2 lps
pec ago2 associated let7a

	A	B	C
	-LPS	4hr LPS	lhr LPS+a-TLR4
	Y	Y	Y
1	1.000000	62.900540	18.151290
2	1.000000	91.624900	37.297340
3	1.000000	50.562640	24.818310
4	1.000000	56.141690	38.204400

FIG EV 2H

Data 91
anti tlr 4 PP2A
anti tlr4 TNFa
phospho ago2 lps
pec ago2 associated let7a
oa liver bhu tnfa
oa liver bhu il1b
PP2A LPG
tnfa LPG

	A	B	C	D	E
	control	1u	10u	1u+anti-TLR4	10u+anti-TLR4
	Y	Y	Y	Y	Y
1	1	1.30	2.55	0.840	1.36
2	1	1.26	2.20	0.670	1.12
3	1	1.14	3.85	0.950	1.35
4	1	2.16	2.02	1.004	1.28
