# Supplementary figures and images for: MicroRNA exporter HuR clears the internalized pathogens by promoting pro‐inflammatory response in infected macrophages
Source: EMBO Mol Med. 2020 Feb 7;12(3):e11011. doi: 10.15252/emmm.201911011 (PMC7059013; doi:10.15252/emmm.201911011)

Figure EV3 Goswami *et al.* Source Data File

EV 3C

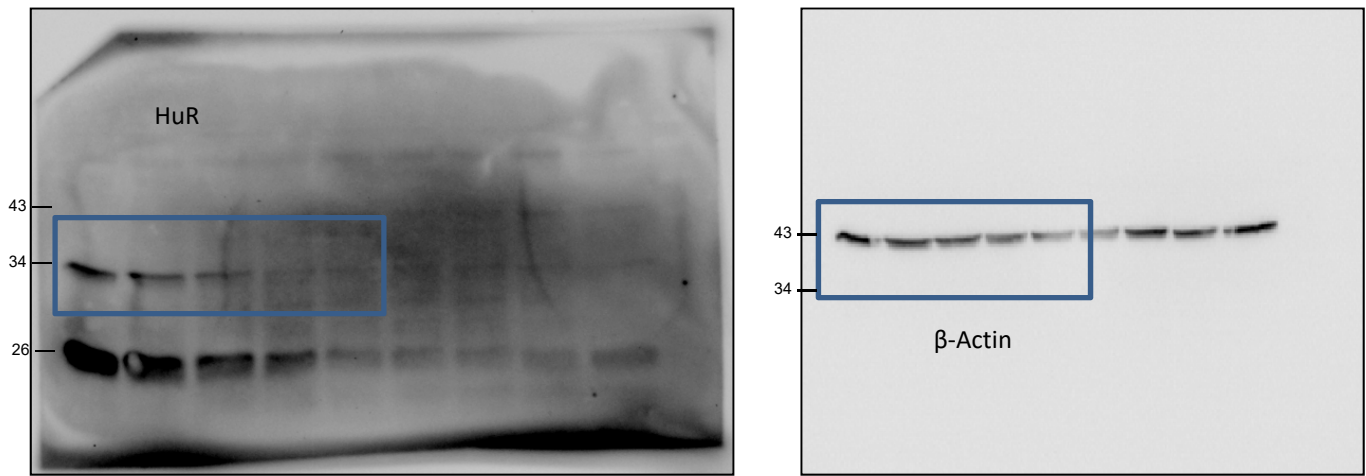

EV 3D

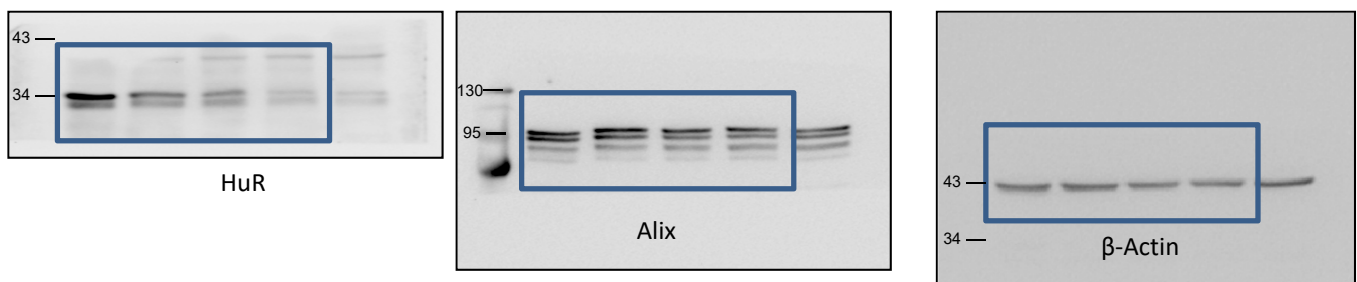

EV 3E

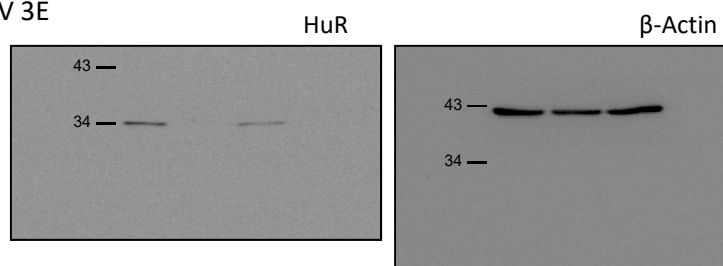

Fig EV 3H

RAW 264.7

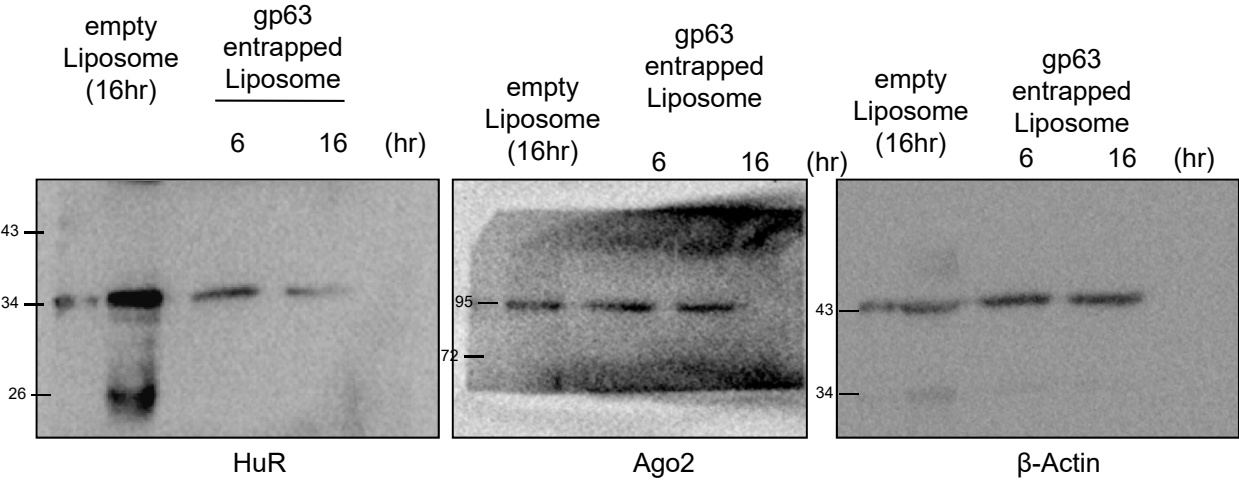

Fig EV 3I

RAW 264.7

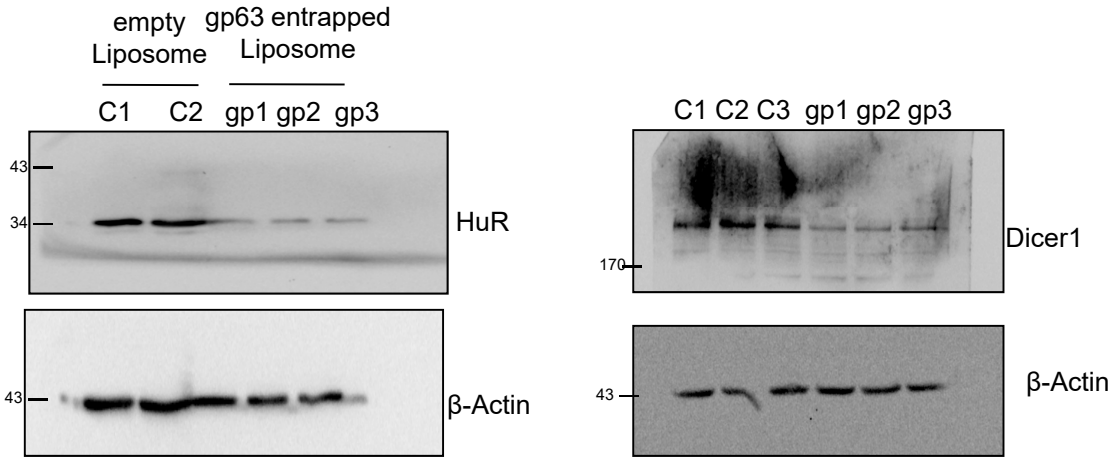

Supplement: Supplementary file 3 — Source Data for Expanded View [file EMMM-12-e11011-s011.zip › Source_Data_for_EV_Figures/Source_Data_for_FigEV3.pdf]
