# Supplementary material for: MicroRNA exporter HuR clears the internalized pathogens by promoting pro‐inflammatory response in infected macrophages
Source: EMBO Mol Med. 2020 Feb 7;12(3):e11011. doi: 10.15252/emmm.201911011 (PMC7059013; doi:10.15252/emmm.201911011)
Supplement: Supplementary file 5 — Source Data for Figure 1 [file EMMM-12-e11011-s003.pdf]

Figure 1 Goswami et al. Source Data File

Fig 1 D

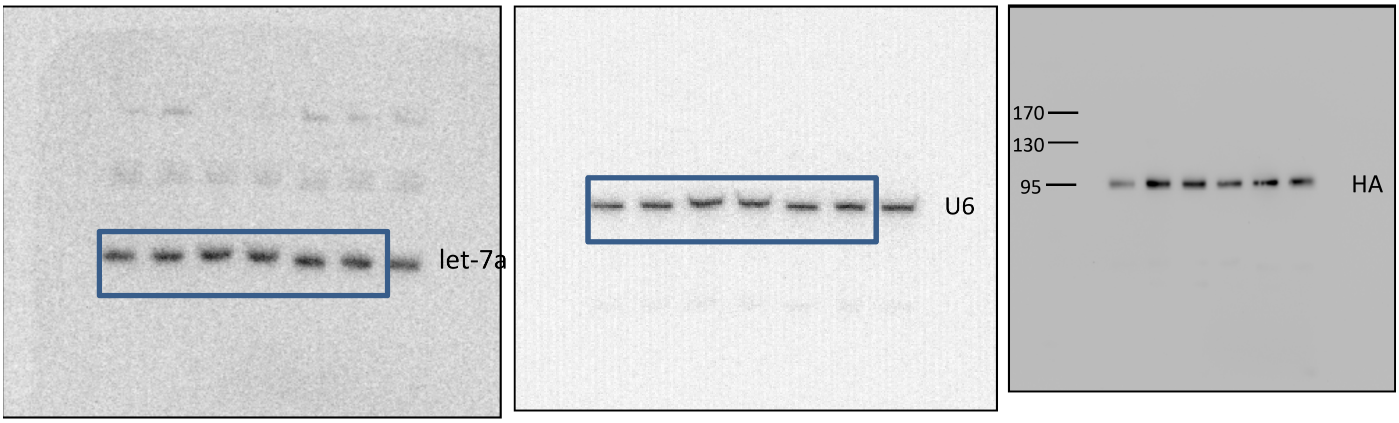

Fig 1 E

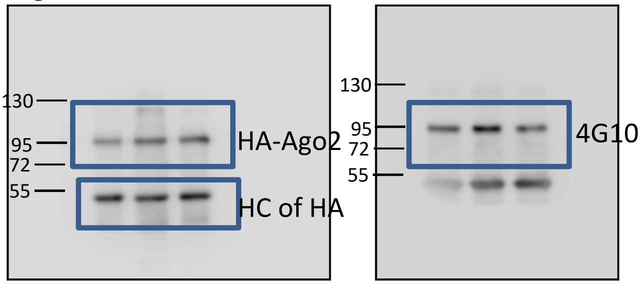

Fig 1 F

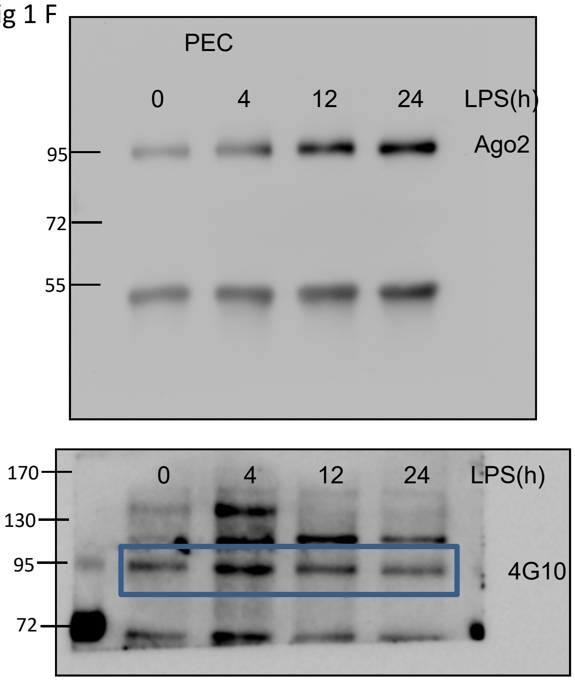

Fig 1 G

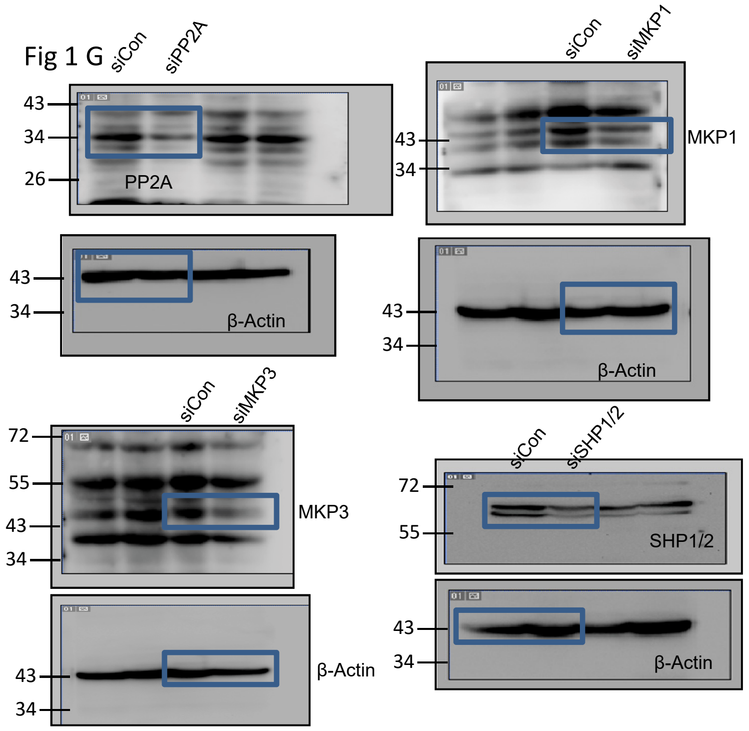

Fig 1 J

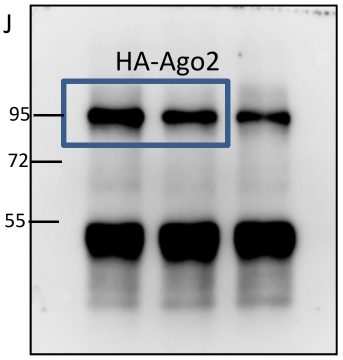

Fig 1 I

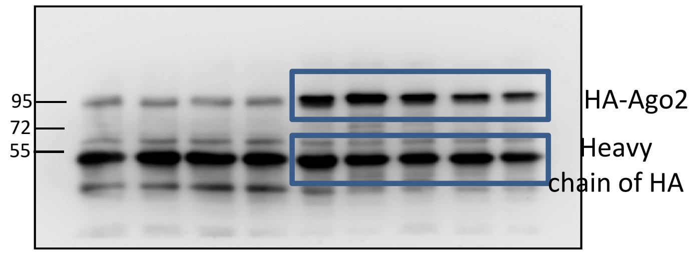

Fig 1 J

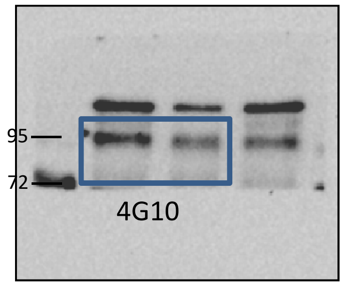

Fig 1 B

- Family
- Data Tables
  - **Figure 1B**
- Info
  - Project info 1
- Results
  - **t test of Figure 1B**
  - **t test of Figure 1B**
  - **t test of Figure 1B**
- Graphs
  - **Figure 1B**
- Layouts

	A	B	C	D
	0hr	3hr	6hr	24hr
	Y	Y	Y	Y
1	1	0.422712	0.542606	0.946184
2	1	0.435500	0.631432	0.833658
3	1	0.468260	0.576060	1.007734
4				
5				
6				

Fig 1 C

- Family
- Data Tables
  - Data 1
  - **Time kinetics TNFa and IL-6 with LPS**
- Info
  - Project info 1
- Results
  - t test of Data 2
  - t test of Data 1
  - t test of Data 1
  - t test of Data 1
  - t test of Data 1
  - t test of Data 1
  - t test of Data 2

		X	A				B		
		X Title	TNFa				IL-6		
		X	A:Y1	A:Y2	A:Y3	A:Y4	B:Y1	B:Y2	B:Y3
1	0hr		1.00	1.00	1.00		1.00	1.00	
2	3hr		59.56	61.18	51.48		28.42	67.77	
3	6hr		33.32	35.20	15.96		22.33	33.55	
4	9hr		4.51	23.03	11.69		5.37	22.63	
5	12hr		6.50	7.99	6.08		3.30	11.34	
6	24hr		5.45	3.88	6.08		3.86	2.94	
7	Title								

Fig 1 E

New ▾ Analyze 1.23 TXT XML

- heat killed il 10 level
- Data 91
- anti tlr 4 PP2A
- anti tlr4 TNFa
- **phospho ago2 lps**
- pec ago2 associated let7a
- oa liver bhu tnfa
- oa liver bhu il1b
- PP2A LPG

	A	B	C
	0hr	4hr	24hr
	Y	Y	Y
1	1	1.400000	0.930000
2	1	1.495622	0.898313
3	1	1.640000	1.140000
4			

Fig 1 H

- Family
- Data Tables
  - Fig 1H
- Info
  - Project info 1
- Results
  - t test of Fig 1H
  - t test of Fig 1H
  - t test of Fig 1H
  - t test of Fig 1H

	A	B	C	D	E	F	G	H	I	J
	Data Set-A	Data Set-B	Data Set-C	Data Set-D	Data Set-E	Data Set-F	Data Set-G	Data Set-H	Data Set-I	Data Set-J
	Y	Y	Y	Y	Y	Y	Y	Y	Y	Y
1	1.000000	1.108900	1.000000	0.885062	1.000000	0.788615	1.000000	0.936161	1.000000	0.872256
2	1.000000	1.044056	1.000000	0.837793	1.000000	0.886203	1.000000	0.941840	1.000000	1.024331
3	1.000000	1.399706	1.000000	0.856994	1.000000	1.310343	1.000000	1.009231	1.000000	1.166889
4	1.000000	1.543749	1.000000	0.937329	1.000000	1.301342	1.000000	1.153963	1.000000	1.293984

Fig 1 I

Data 9
tnfa_siPP2A
IL-6_siPP2A
Ld internalization
IL-10_siPP2A
siphosphatases
TNFa_tolerance
siPTPA_IP_Let7a_146a
dox_RNP Biogenesis
Tolerance

	A	B	C	D	E
	siCon	siPP2A	siMKP1	siMKP6	siSHP1/2
	Y	Y	Y	Y	Y
1	1	0.303900	1.059462	0.871564	1.113121
2	1	0.600086	1.912801	2.458110	3.048232
3	1	0.412109	1.720614	2.927357	1.839581
4			0.426380		

Fig 1 K

Family
Data Tables
Data 1
siPP2A_IP_Let7a_miR155_146
miR155
miR122
siC siP
LPS
Data 8
Data 9
tnfa_siPP2A

	A	B	C	D	E	F	G	H
	siCon_LPS	siPP2A_LPS	Title	siCon_LPS	siPP2A_LPS	Title	siCon_LPS	siPP2A_LPS
	Y	Y	Y	Y	Y	Y	Y	Y
1	1.0000	0.393902		1.000000	0.684919		1.000000	0.504269
2	1.0000	0.482474		1.000000	0.565598		1.000000	0.493132
3	1.0000	0.373540		1.000000	0.571827		1.000000	0.344156
4	1.0000	0.266246		1.000000	0.500632		1.000000	0.647297
5	1.0000	0.659891		1.000000	0.536494			
