# Supplementary material for: MicroRNA exporter HuR clears the internalized pathogens by promoting pro‐inflammatory response in infected macrophages
Source: EMBO Mol Med. 2020 Feb 7;12(3):e11011. doi: 10.15252/emmm.201911011 (PMC7059013; doi:10.15252/emmm.201911011)
Supplement: Supplementary file 6 — Source Data for Figure 2 [file EMMM-12-e11011-s004.pdf]

Figure 2 Goswami et al. Source Data File

Fig 2 B

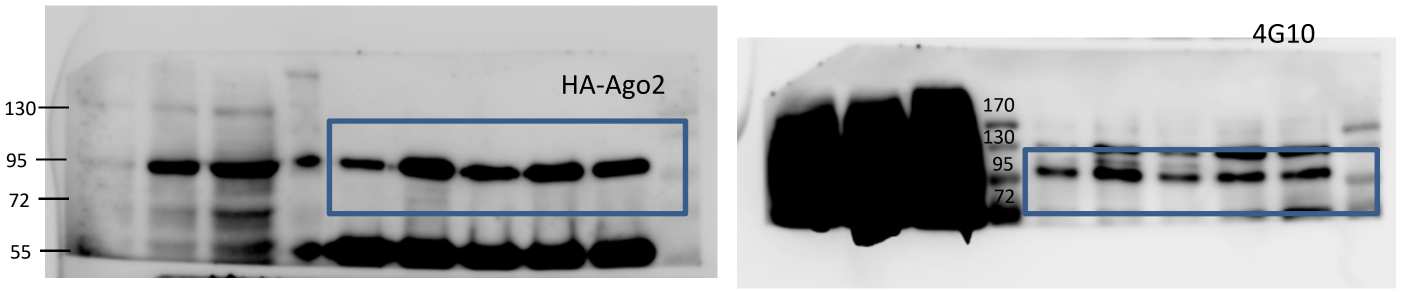

Fig 2 D

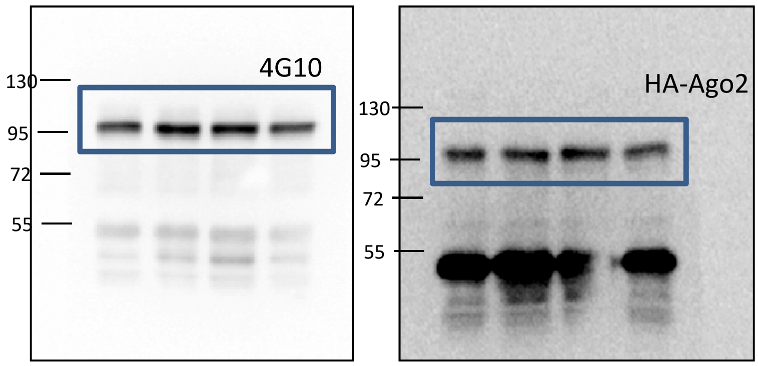

Fig 2 F

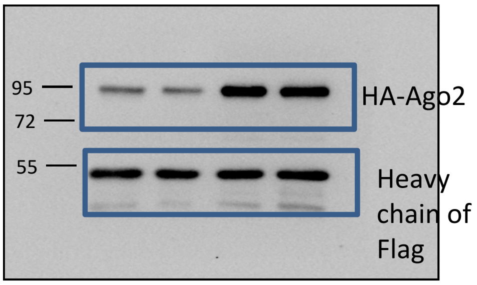

Fig 2 H

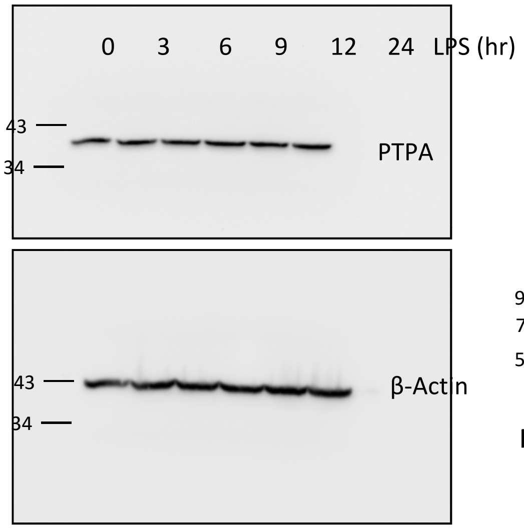

Fig 2 I

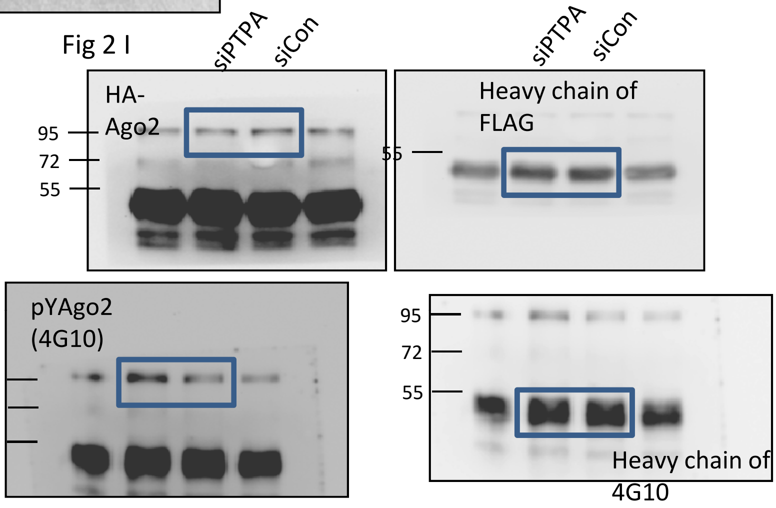

Fig 2 I

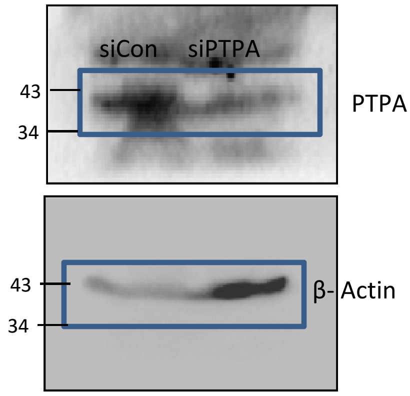

Fig 2 H

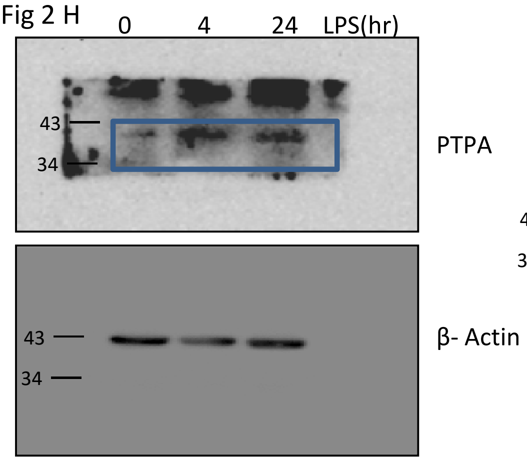

Fig 2 K

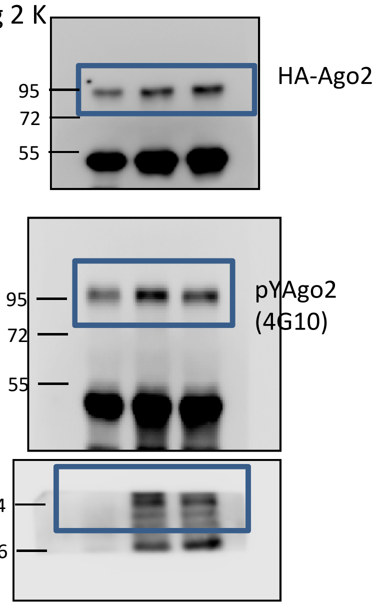

Fig 2 C

- Family
- Data Tables
  - **Data 2C**
- Info
  - Project info 1
- Results
  - **t test of Data 2C**
- Graphs
  - **Data 2C**
- Layouts

	A	B	C	D
	Data Set-A	Data Set-B	Data Set-C	Data Set-D
	Y	Y	Y	Y
1	1	1.426685	0.631418	0.679305
2	1	1.696200	0.613010	0.695335
3	1	1.474422	0.665683	0.793478
4				
5				
6				

Fig 2 F

- HA_HUR_LD_Cytokine
- HA_HUR_LD_PP2A subunit
- pp2a_ag83_bhu_sg
- HAHUR_PP2A subunits_no LPS
- Data 63
- Data 64
- TNFa_+/-LPS_HAHUR
- IL1B_+/-LPS_HAHUR
- HAHUR_BHU_%infected
- [illegible]

	A	B	C	D
	14hr DOX	24hr DOX	14hr DOX	24hr DOX
	Y	Y	Y	Y
1	1.000000	4.505352	1.000000	1.090000
2	1.000000	5.189962	1.000000	1.020000
3	1.000000	10.450000	1.000000	1.360000
4	1.000000	8.230000	1.000000	1.520000

Fig 2 J

- Data 9
- tnfa_siPP2A
- IL-6_siPP2A
- Ld internalization
- IL-10_siPP2A
- siphosphatases
- TNFa_tolerance
- **siPTPA_IP_Let7a_146a**

	A	B	C	D	E
	siCon	siPTPA	Title	siCon	siPTPA
	Y	Y	Y	Y	Y
1	1	0.65		1	0.59
2	1	0.60		1	0.54
3	1	0.45		1	0.63
