# Supplementary material for: MicroRNA exporter HuR clears the internalized pathogens by promoting pro‐inflammatory response in infected macrophages
Source: EMBO Mol Med. 2020 Feb 7;12(3):e11011. doi: 10.15252/emmm.201911011 (PMC7059013; doi:10.15252/emmm.201911011)
Supplement: Supplementary file 7 — Source Data for Figure 3 [file EMMM-12-e11011-s005.pdf]

Figure 3 Goswami et al. Source Data File

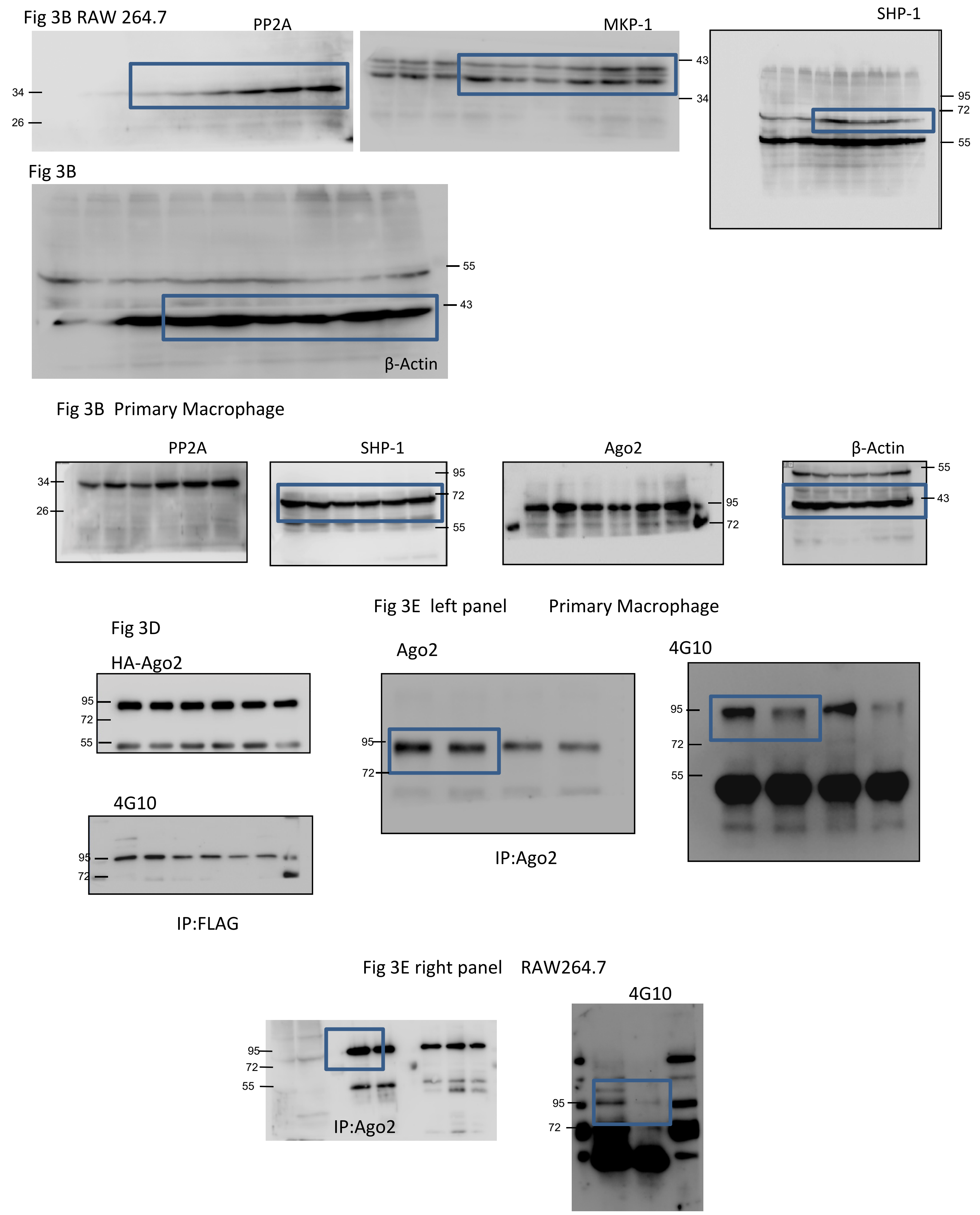

Fig 3 continued

Fig 3F

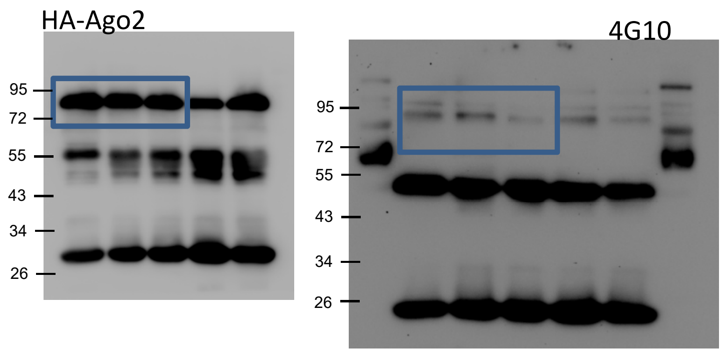

Fig 3F

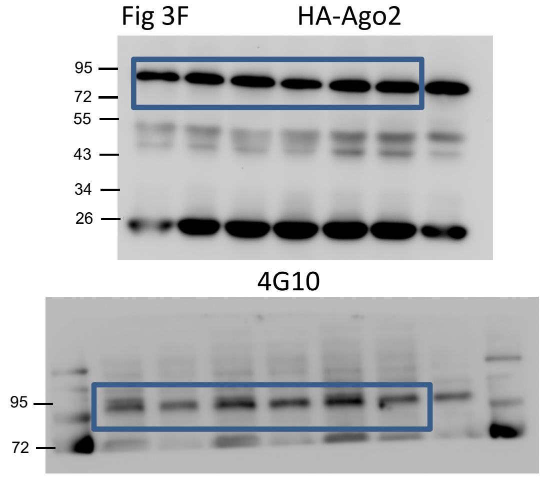

Fig 3J

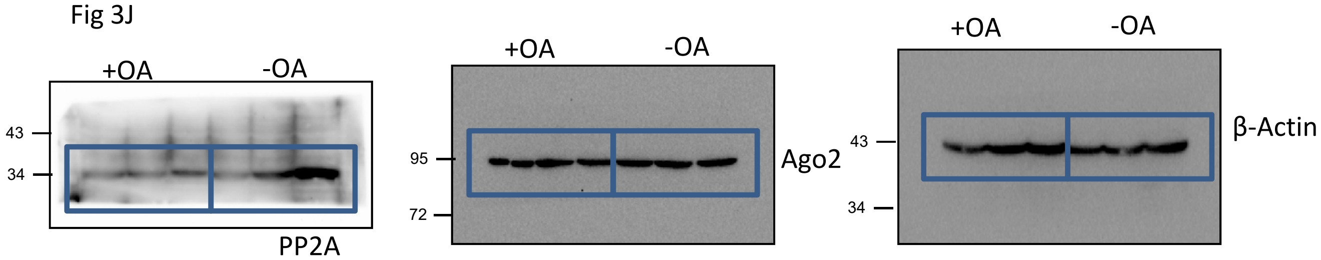

Fig 3K

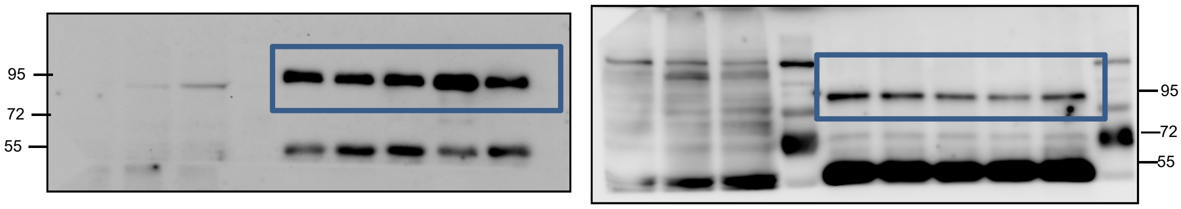

Fig 3K

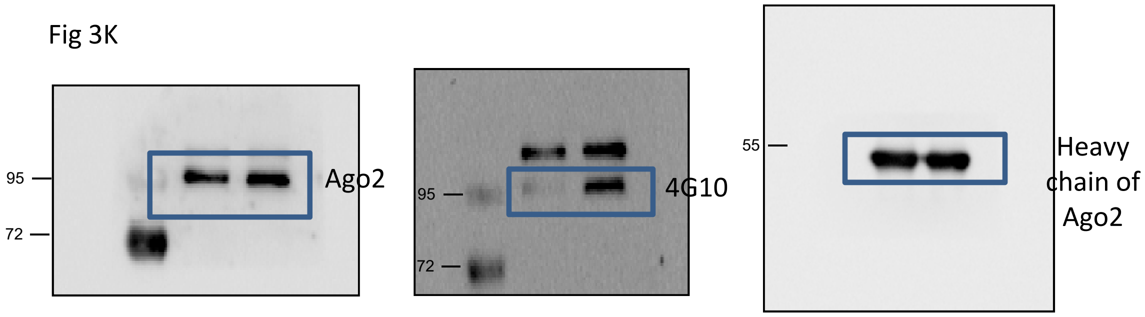

Fig 3L

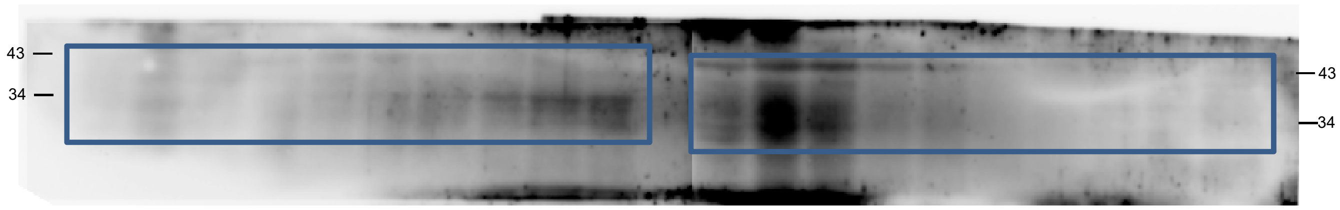

Fig 3C

Family
Data Tables
cytokine mRNA levels RAW
Info
Project info 1
Results
t test of cytokine mRNA le
t test of cytokine mRNA le
t test of cytokine mRNA le
Graphs
cytokine mRNA levels RAW
Layouts

	A	B	C	D	E	F
	Data Set-A	Data Set-B	Data Set-C	Data Set-D	Data Set-E	Data Set-F
	Y	Y	Y	Y	Y	Y
1	1.000000	2.418997	1	0.658917	1	0.648439
2	1.000000	2.592667	1	0.287404	1	0.769282
3	1.000000	1.610246	1	0.220904	1	0.526538
4						
5						
6						
7						

Fig 3C

tolerance _il1b
tolerance_il10
yf_IL-6_pp2a
Data 31
Data 32
WT/YF_pp2A_IL-10
PEC_LD_cytokine
PEC_OA_IL6
PEC_OA_IL10
il-1b siPP2A
siPTPA_Phos assay 4g10
siPP2A_24 lPS_Cellular_7A_146a

	A	B	C	D	E	F
	0hr	24hr	0hr	24hr	0hr	24hr
	Y	Y	Y	Y	Y	Y
1	1	1.396087	1	0.251085	1.000000	0.522353
2	1	1.254841	1	0.481268	1.000000	0.459733
3	1	1.635908	1	0.628124	1.000000	0.487950
4						
5						
6						

Fig 3E right panel

pp2a c ld lm infect
pp2a subunit +- LPS
Ld mathematical
Data 81
Data 82
IP_LD_Ago2
Ago2 associated Let7a LPS timepoint
Data 85

	A	B	C
	0hr Ld	4hr Ld	Title
	Y	Y	Y
1	1.000000	2.534692	
2	1.000000	3.201879	
3	1.000000	2.141043	

Fig 3G

heat killed il 10 level
Data 91
anti tlr 4 PP2A
anti tlr4 TNFa
phospho ago2 lps
pec ago2 associated let7a
oa liver bhu tnfa
oa liver bhu il1b

	A	B	C
	Bead Control	-Ld	+Ld
	Y	Y	Y
1	1	0.97	0.36
2	1	1.08	0.57
3	1	0.98	0.30

Fig 3I

Data 72
LPS mathematical
Data 74
Data 75
ld mathematical
sicon sipp2a_ld
pp2a c ld lm infect
pp2a subunit +- LPS

	A	B	C	D
	Data Set-A	Data Set-B	Data Set-C	Data Set-D
	Y	Y	Y	Y
1	1.000000	4.341569	0.776223	3.113135
2	1.000000	6.455529	1.000000	2.897917
3	1.000000	6.272841	1.000000	1.718596
