# Supplementary material for: MicroRNA exporter HuR clears the internalized pathogens by promoting pro‐inflammatory response in infected macrophages
Source: EMBO Mol Med. 2020 Feb 7;12(3):e11011. doi: 10.15252/emmm.201911011 (PMC7059013; doi:10.15252/emmm.201911011)
Supplement: Supplementary file 8 — Source Data for Figure 4 [file EMMM-12-e11011-s006.pdf]

Figure 4 Goswami et al. Source Data File

Fig 4 A IL-10 RAW264.7

- Family
- Data Tables
  - Data 1
- Info
  - Project info 1
- Results
  - t test of Data 1
  - t test of Data 1

	A	B	C	D
	Data Set-A	Data Set-B	Data Set-C	Data Set-D
	Y	Y	Y	Y
1	1.000000	1.537729	0.781443	0.928040
2	1.000000	2.418997	1.694886	0.757679
3	1.000000	1.596018	1.000000	0.541777

Fig 4 A IL-10 PEC

- tolerance _il1b
- tolerance_il10
- yf_IL-6_pp2a
- Data 31
- Data 32
- WT/YF_pp2A_IL-10
- PEC_LD_cytokine
- PEC_OA_IL6

	A	B	C	D
	Data Set-A	Data Set-B	Data Set-C	Data Set-D
	Y	Y	Y	Y
1	1.000000	4.207189	1.000000	0.565335
2	1.000000	2.367449	1.000000	0.851650
3	1.000000	3.265961	1.000000	1.018774

Fig 4 A IL-6 RAW264.7

- Family
- Data Tables
  - Data 1
- Info
  - Project info 1
- Results
  - t test of Data 1
- Graphs

	A	B	C	D
	Data Set-A	Data Set-B	Data Set-C	Data Set-D
	Y	Y	Y	Y
1	1.000000	0.648439	1.542318	2.038601
2	1.000000	0.769282	1.241749	1.977278
3	1.000000	0.512623	0.586573	1.429170

Fig 4 A IL-6 PEC

- tolerance _il1b
- tolerance_il10
- yf_IL-6_pp2a
- Data 31
- Data 32
- WT/YF_pp2A_IL-10
- PEC_LD_cytokine
- **PEC_OA_IL6**
- PEC_OA_IL10

	A	B	C	D
	Data Set-A	Data Set-B	Data Set-C	Data Set-D
	Y	Y	Y	Y
1	1.000000	0.535887	1.000000	1.420151
2	1.000000	0.445141	1.000000	1.907477
3	1.000000	0.793151	1.000000	1.420763
4	1.000000	0.609205		

Fig 4 C left panel

- siPP2A_IP_Let7a_miR155_146a
- miR155
- miR122
- siC siP
- LPS
- Data 8
- Data 9
- tnfa_siPP2A
- IL-6_siPP2A
- **Ld internalization**
- IL-10_siPP2A
- siphosphatases
- TNFa_tolerance
- siPTPA_IP_Let7a_146a
- dox_RNP Biogenesis
- Tolerance
- il-1b siPTPA
- IL-6 siPTPA
- siPTPA_IP_IL1b_il6_IL-10
- dox_biogenesis_122
- dox_biogenesis_let7a
- Data 24
- il10_siPTPA
- ros production
- tolerance _il1b
- tolerance_il10
- yf_IL-6_pp2a
- Data 31
- Data 32
- WT/YF_pp2A_IL-10
- PEC_LD_cytokine
- PEC_OA_IL6
- PEC_OA_IL10

	A	B
	siCon	siPP2A
	Y	Y
1	3.00	2.00
2	4.00	1.00
3	3.00	1.00
4	2.00	2.00
5	3.00	2.00
6	3.00	1.00
7	4.00	3.00
8	3.00	2.00
9	3.00	1.00
10	2.00	3.00
11	2.00	2.00
12	4.00	2.00
13	3.00	1.00
14	3.00	1.00
15	4.00	2.00
16	3.00	1.00
17	3.00	1.00
18	4.00	1.00
19	3.00	2.00
20	3.00	1.00
21		

Fig 4 C Right panel

- Ago2 associated Let7a LPS timepoint
- Data 85
- Data 86
- pp2a c subunit heat killed
- l major cytokine tnf il10
- heat killed tnfa level
- heat killed il 10 level
- Data 91

	A	B
	siCon_LPS	siPP2A_LPS
	Y	Y
1	1	0.6400
2	1	0.5800
3	1	0.4548

Fig 4 F Left panel

- Data 43
- dox_cellular
- dox _IP
- **WT_YF_OA_LD**
- pci hahuR_PP2A levels
- Data 49
- Data 50

	WT	YF	WT	YF	WT	YF
	Y	Y	Y	Y	Y	Y
1	1	0.789400	1	0.760094	1	1.708484
2	1	0.374507	1	0.663194	1	1.581001
3	1	0.914635	1	0.629036	1	1.425150
