# Supplementary material for: MicroRNA exporter HuR clears the internalized pathogens by promoting pro‐inflammatory response in infected macrophages
Source: EMBO Mol Med. 2020 Feb 7;12(3):e11011. doi: 10.15252/emmm.201911011 (PMC7059013; doi:10.15252/emmm.201911011)
Supplement: Supplementary file 9 — Source Data for Figure 5 [file EMMM-12-e11011-s007.pdf]

Figure 5 Goswami et al. Source Data File

Fig 5G

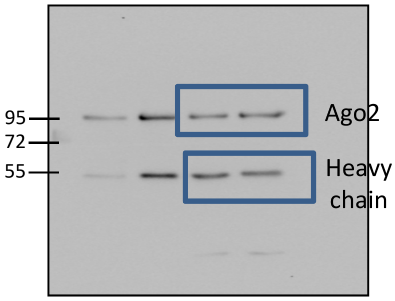

Fig 5H

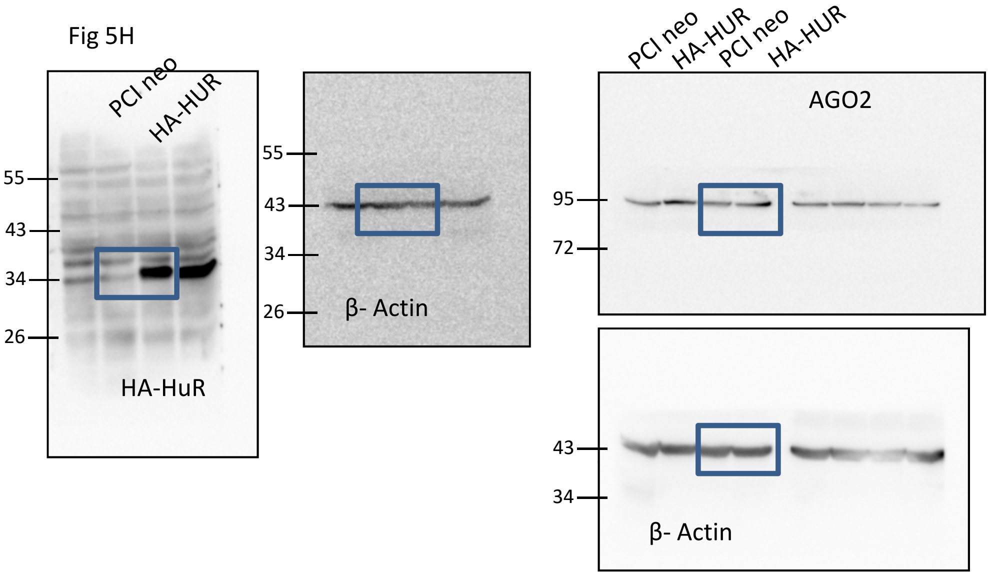

Fig 5J

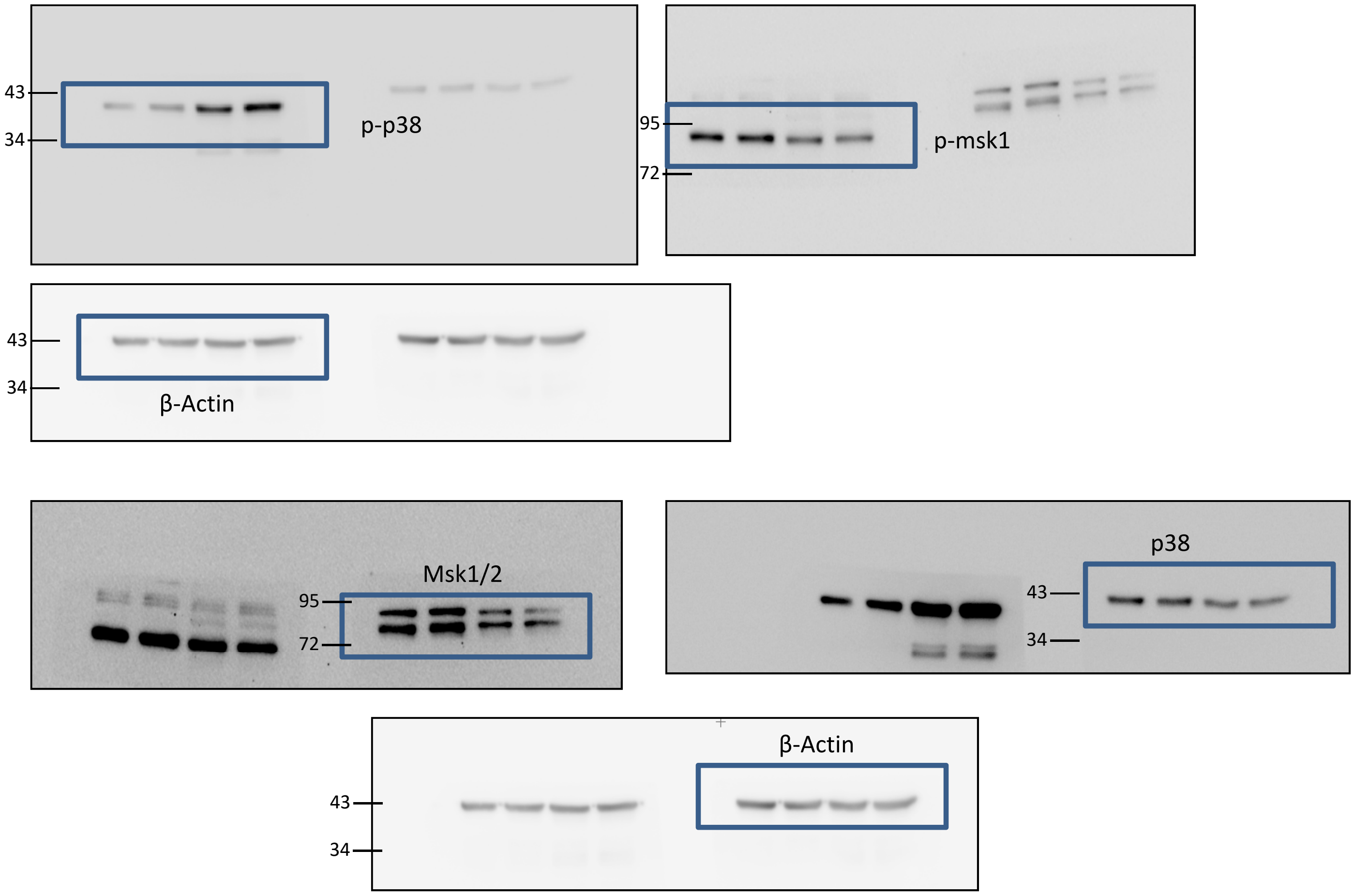

Fig 5K upper left panel

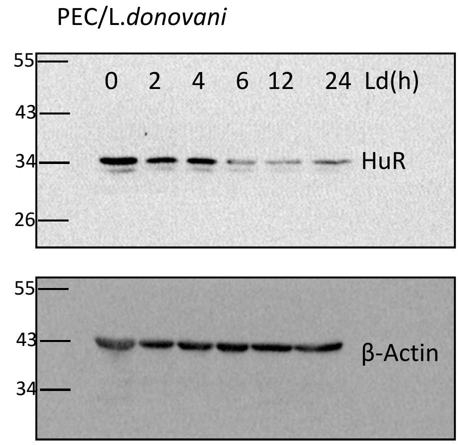

Fig 5K upper right panel

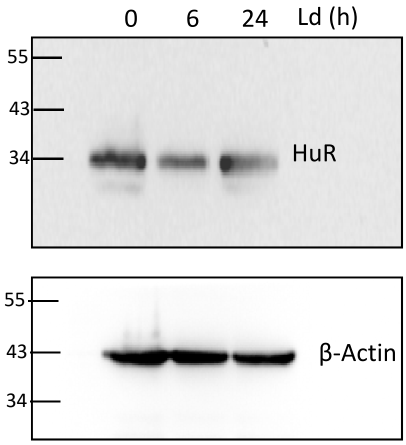

Fig 5K lower right panel

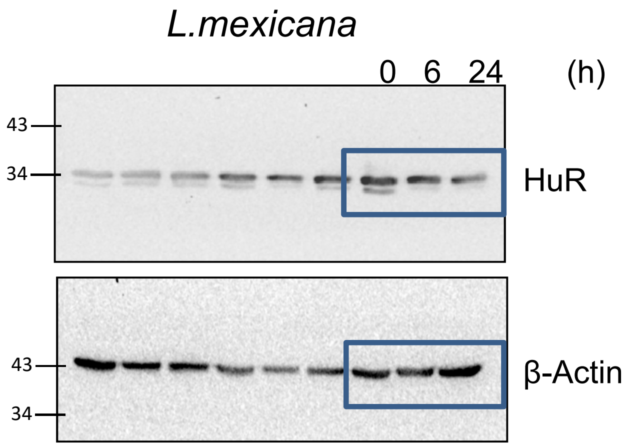

Fig 5K lower left panel

L.major

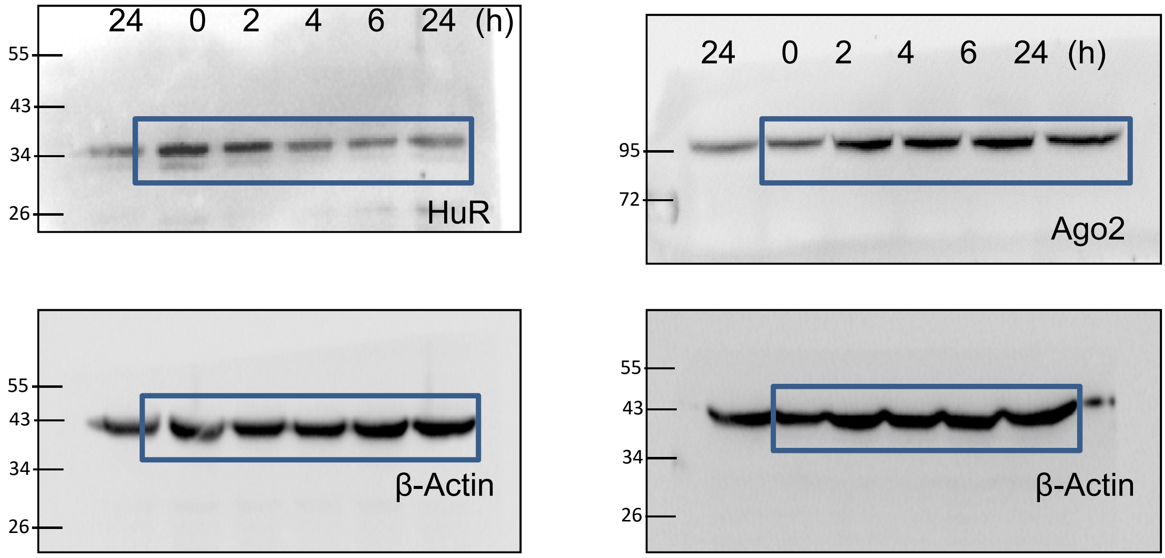

Fig 5L

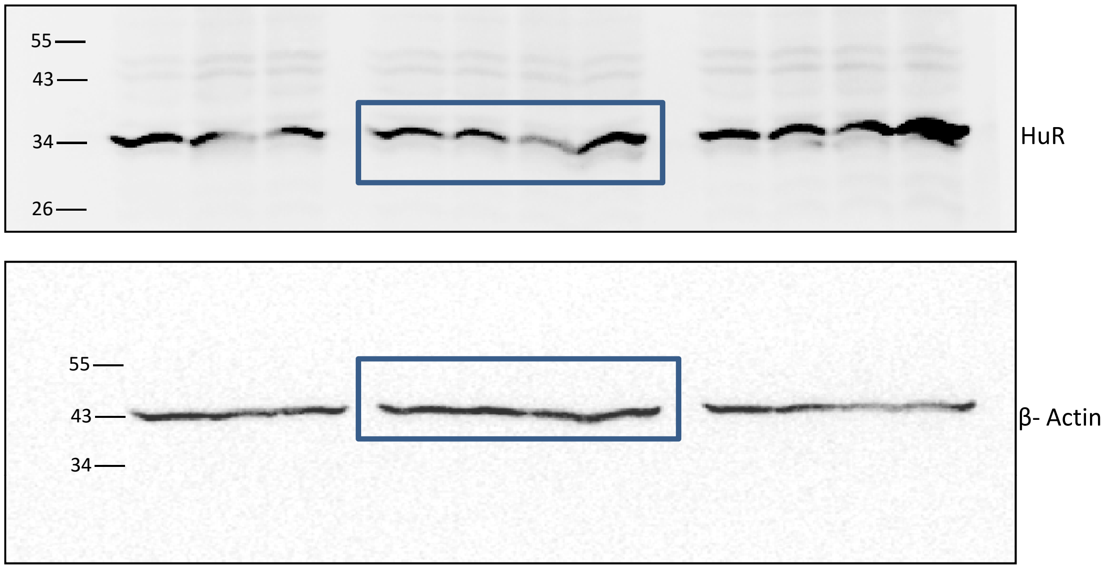

Fig 5M input

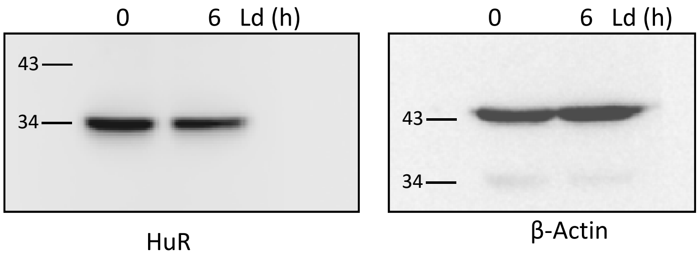

Fig 5M gradient

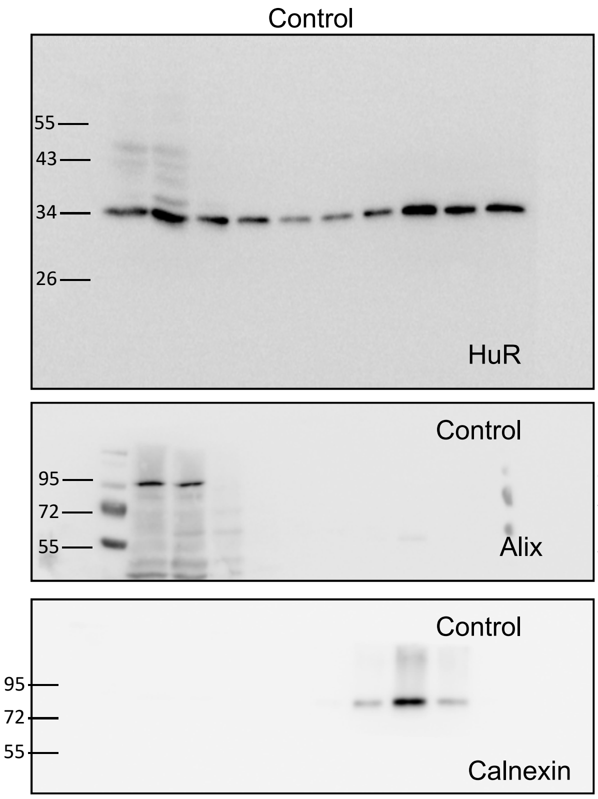

Fig 5N

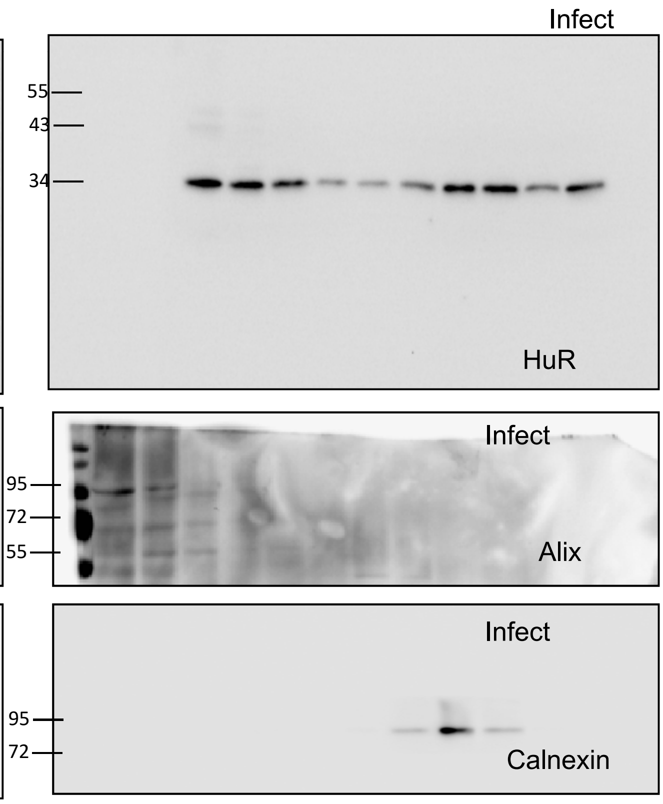

Fig. 5C

let-7a Wo LPS	HuR bound W LPS	miR-155 WoLPS	HuR bound W LPS
1	1.51	1	8.66
1	1.93	1	6.31
1	1.79	1	4.74
1	1.79	1	6.31

Fig. 5E

TNF-a			IL-6		
Wo LPS	W LPS	WLPS +GW	Wo LPS	W LPS	W LPS+GW4869
1	18.46	3.11	1	20.09	2
1	21.8	0.99	1	17.52	1
1	18.5	1.62	1	27.23	1.4
1	17.29	1.91	1	21.84	1.7
1	18.48	1.765	1	20.965	1.55

Fig. 5F

TNFa		IL-6		IL-1B		miR-155	
con siRNA	SiHuR	ConsiRNA	SiHuR	ConsiRNA	SiHuR	ConsiRNA	SiHuR
1	0.7	1	0.39	1	0.47	1	23.66
1	0.42	1	0.57	1	0.55	1	37.6
1	0.45	1	0.7	1	0.68	1	39.1
1	0.45	1	0.57	1	0.55	1	37.6

Fig 5 G

HAHUR_PP2A subunits_no LPS
Data 63
Data 64
TNFa_+/-LPS_HAHUR
IL1B_+/-LPS_HAHUR
HAHUR_BHU_%infected
siHUR_6hr LPS
4G10 level in fraction Ld inf

	A	B
	siCON_6hr LPS	siHuR_6hr LPS
	Y	Y
1	1	2.445110
2	1	2.312739
3	1	2.432898

Fig 5 I left panel

parasite count oa 2 24 hr
lps phosphatases
Data 106
wt yf ld internalized
hahur ag83 internalized
pci HAHUR_LPS_TNFa
pp2a phosphatase assay
Data 111

	A	B	C	D
	PCI_Neo-LPS	HA_HuR-LPS	PCI_Neo+LPS	HA_HuR+LPS
	Y	Y	Y	Y
1	1.00	1.80	3.18	3.81
2	1.00	2.53	2.85	4.30
3	1.00	3.10	3.14	5.42

FIG 5 I right panel

amily
ata Tables
HA_HUR_BHU569
HA_HUR_BHU569_OKA
neo_bhu_oka final
HAHUR_OKA_BHU_repeat0305
TNF_Ha_hur-OA

	A	B	C	D
	neo-LPS	HuR-LPS	neo+LPS	HuR+LPS
	Y	Y	Y	Y
1	662.666700	1536.000000	2642.667000	3574.333000
2	904.333300	2202.667000	2699.333000	3859.333000
3	931.000000	1812.667000	2974.333000	3334.333000

Fig 5 L lower panel

Family
Data Tables
mg132
ha hur
Info
Project info 1
Results
t test of mg132

	A	B
	- mg132	+mg132
	Y	Y
1	1	1.707241
2	1	1.773174
3	1	1.501426
