# Supplementary material for: MicroRNA exporter HuR clears the internalized pathogens by promoting pro‐inflammatory response in infected macrophages
Source: EMBO Mol Med. 2020 Feb 7;12(3):e11011. doi: 10.15252/emmm.201911011 (PMC7059013; doi:10.15252/emmm.201911011)
Supplement: Supplementary file 10 — Source Data for Figure 6 [file EMMM-12-e11011-s008.pdf]

Figure 6 Goswami et al. Source Data File

Fig 6B

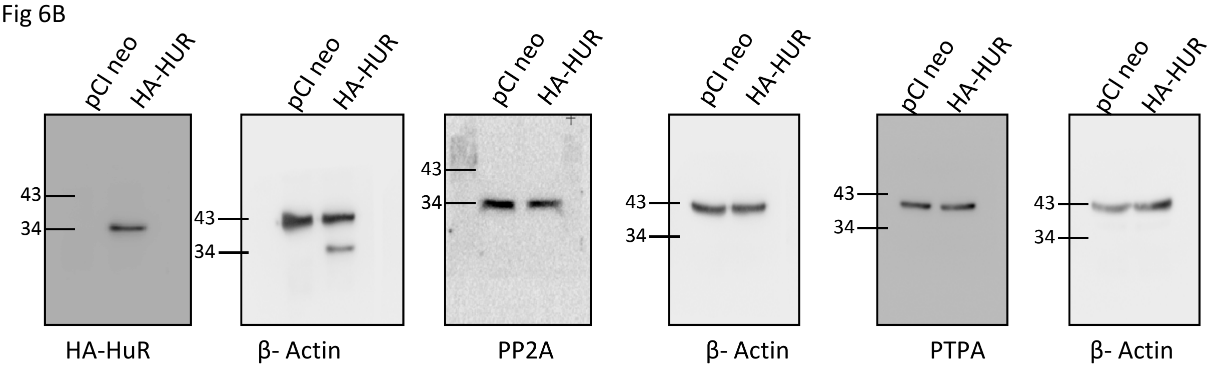

Figure 6C

Data 43
dox_cellular
dox _IP
WT_YF_OA_LD
pci hahuR_PP2A levels
Data 49
Data 50
Data 51

	A	B	C	D	E	F
	Data Set-A	Data Set-B	Data Set-C	Data Set-D	Data Set-E	Data Set-F
	Y	Y	Y	Y	Y	Y
1	1	0.419748	1	0.432554	1	0.590380
2	1	0.431751	1	0.477737	1	0.578143
3	1	0.494623	1	0.516795	1	0.729895

Figure 6D

IL1B_+/-LPS_HAHUR
HAHUR_BHU_%infected
siHUR_6hr LPS
4G10 level in fraction Ld inf
LPS recycling +- OA
Data 72
LPS mathematical
Data 74
Data 75
ld mathematical
sicon sipp2a_ld
pp2a c ld lm infect

Table format: XY		X	A	B	C	D
		Timepoint LPS	miRNA activity	IL-6	PP2A level	HuR Level
		X	Y	Y	Y	Y
1	Title	0	1.000000	1.000000	0.714855	0.123
2	Title	3	0.337306	51.482150	0.956421	0.456
3	Title	6	0.363399	15.963080	0.985034	0.355
4	Title	9	0.650000	11.688360	1.010815	0.899
5	Title	12	0.934876	6.082512	0.996961	0.910
6	Title	24	1.062099	6.082512	1.000000	1.000
