# Supplementary material for: MicroRNA exporter HuR clears the internalized pathogens by promoting pro‐inflammatory response in infected macrophages
Source: EMBO Mol Med. 2020 Feb 7;12(3):e11011. doi: 10.15252/emmm.201911011 (PMC7059013; doi:10.15252/emmm.201911011)
Supplement: Supplementary file 11 — Source Data for Figure 7 [file EMMM-12-e11011-s009.pdf]

Figure 7 Goswami et al. Source Data File

Fig 7D

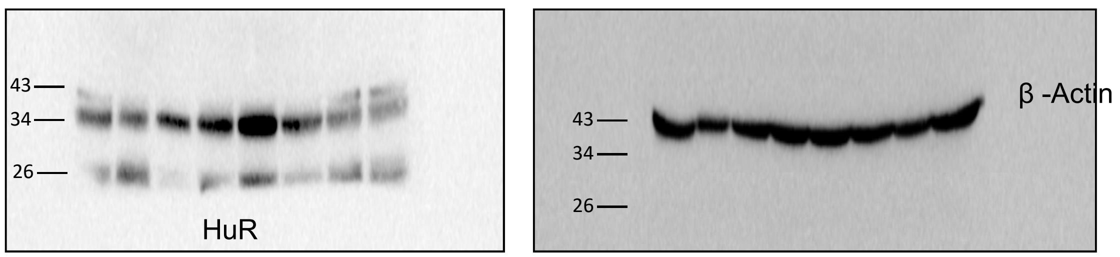

Fig 7E

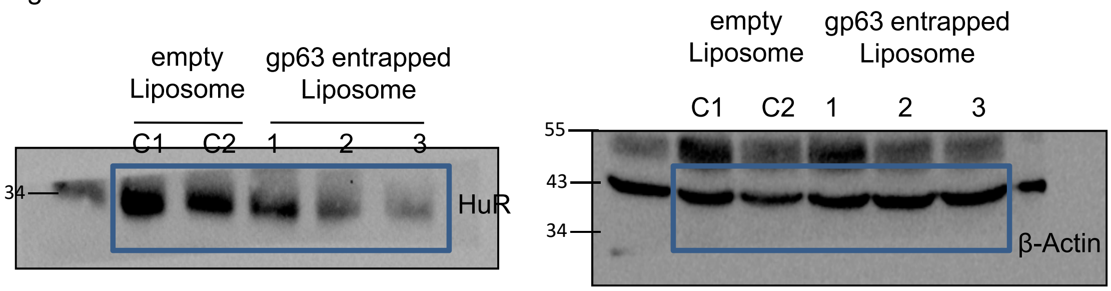

Fig 7F

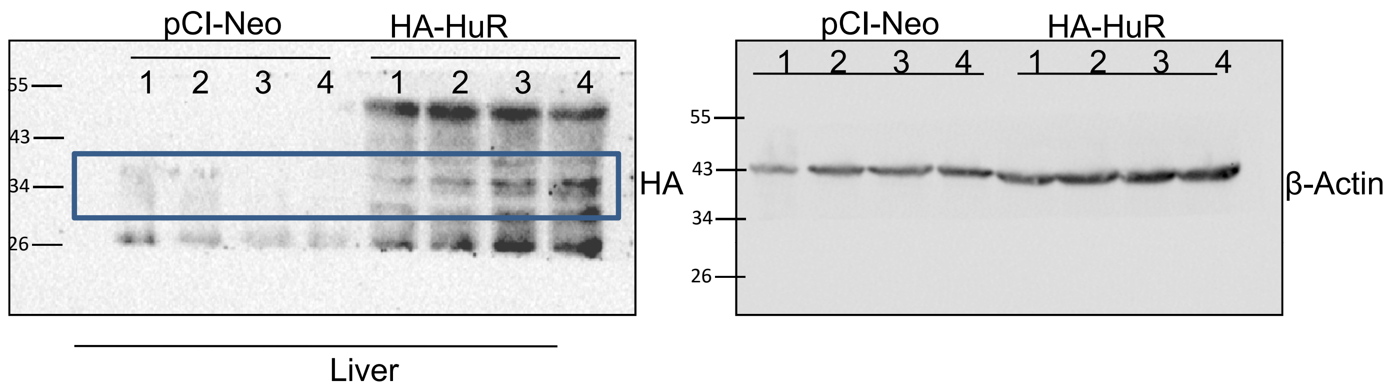

Fig 7J

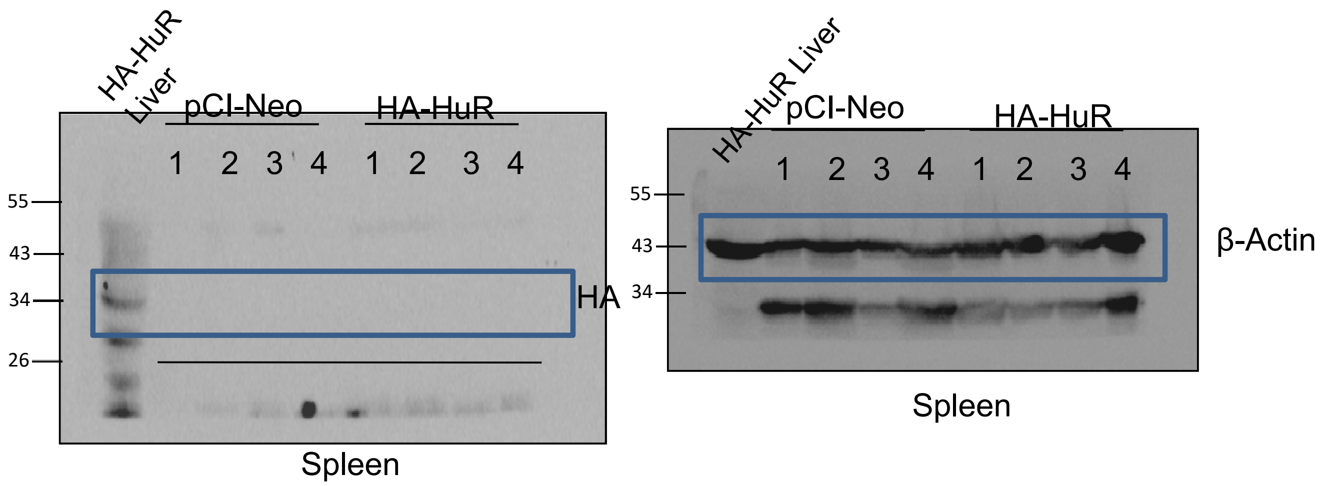

Data 85
Data 86
pp2a c subunit heat killed
l major cytokine tnf il10
heat killed tnfa level
heat killed il 10 level
Data 91
anti tlr 4 PP2A
anti tlr4 TNFa
phospho ago2 lps
pec ago2 associated let7a
oa liver bhu tnfa
oa liver bhu il1b
PP2A LPG
tnfa LPG
oa liver bhu ld count
parasite count oa 2 24 hr
lps phosphatases
Data 106
wt yf ld internalized
hahur ag83 internalized
pci HAHUR_LPS_TNFa
pp2a phosphatase assay
Data 111
Data 112
PP2A subunits +- LPS
Data 114
Data 115
Data 116
Data 117
-/+ pd98059
Info
Project info 1

	A	B
	Data Set-A	Data Set-B
	Y	Y
1	3	1
2	6	2
3	4	1
4	5	0
5	2	2
6	5	1
7	6	1
8	4	0
9	4	2
10	3	1
11	5	1
12	7	3
13	6	2
14	4	1
15	6	2
16	6	2
17	7	1
18	3	1
19	5	2
20	4	2
21	4	3

DOX_OA
Data 56
LPS_Let7a_Ago2 associated_Northe
LD infected fraction cell_HA_HUR
HA_HUR_LD_Cytokine
HA_HUR_LD_PP2A subunit
pp2a_ag83_bhu_sg

	A	B	C	D	E	F
	PCI_Neo	HA_HuR	PCI_Neo	HA_HuR	PCI_Neo	HA_HuR
	Y	Y	Y	Y	Y	Y
1	1	5.122398	1	4.853125	1	0.420236
2	1	7.388568	1	3.316952	1	0.422358
3	1	7.639124	1	3.878653	1	0.503094

LD count
Ld count dot plot liver
TNFa dot plot
IL10 dot plot
HuR band quantify
Data 9
Data 10
Data 11
Data 12
Ld count in spleen

	0days LD	10days LD	35days LD
	Y	Y	Y
1	1.000000	0.694784	0.563615
2	1.000000	0.401811	0.503704
3	1.000000	0.373968	0.484763
4	1.000000	0.609549	0.501120
5	0.839059	0.591895	0.454786

Fig 7G

Family
Data Tables
- TNFa
- IL-10
- LD count
- **Ld count dot plot liver**
- TNFa dot plot
- IL10 dot plot
- HuR band quantify
- Data 9
- Data 10

	A	B
	PCI_Neo	HA_HuR
	Y	Y
1	8.446000	12.680500
2	9.510000	11.197000
3	9.579333	10.885000
4	9.228000	12.550670
5	9.615000	10.276000

Fig 7H

Family
Data Tables
- TNFa
- IL-10
- LD count
- Ld count dot plot liver
- **TNFa dot plot**
- IL10 dot plot
- HuR band quantify
- Data 9
- Data 10
- Data 11

Table format: **Column**	A	B
	PCI_Neo	HA_HuR
	Y	Y
1	15.229000	14.266000
2	15.751000	12.916670
3	15.624170	14.749000
4	15.212330	14.793000
5	15.474330	14.382670
6	15.006670	

Fig 7I

Family
Data Tables
- TNFa
- IL-10
- LD count
- Ld count dot plot liver
- TNFa dot plot
- **IL10 dot plot**
- HuR band quantify
- [illegible]

Table format: **Column**	A	B
	PCI_Neo	HA_HuR
	Y	Y
1	16.092000	16.766000
2	16.416330	17.852000
3	16.274330	19.054000
4	16.515170	17.411000

Fig 7K

- Data 12
- Ld count in spleen
- Data 14
- Data 15
- HAHUR_BHU
- **spleen tnfa pci hahur**
- spleen il-10 pci hur
- spleen ld count pci hur
- tnfa hahur ag83
- [illegible]

Table format: **Column**	A	B
	pCI-neo	HA-HuR
	Y	Y
1	1.000000	1.536875
2	1.000000	0.801996
3	1.000000	0.756983
4	1.000000	1.180993
