# Supplementary material for: MicroRNA exporter HuR clears the internalized pathogens by promoting pro‐inflammatory response in infected macrophages
Source: EMBO Mol Med. 2020 Feb 7;12(3):e11011. doi: 10.15252/emmm.201911011 (PMC7059013; doi:10.15252/emmm.201911011)
Supplement: Supplementary file 12 — Source Data for Figure 8 [file EMMM-12-e11011-s010.pdf]

Figure 8 Goswami et al. Source Data File

Fig 8D

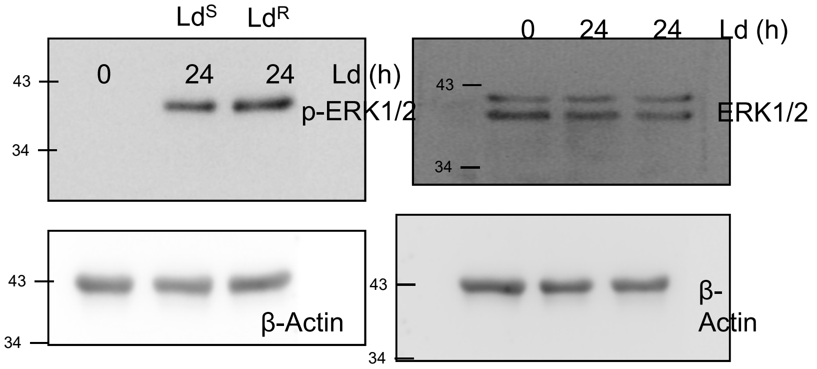

Fig 8D

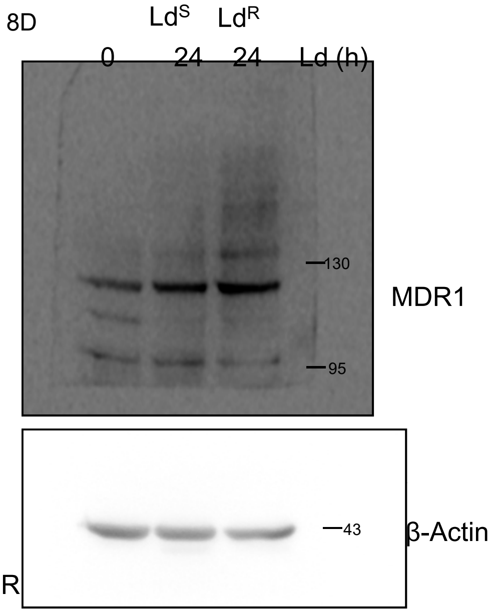

Fig 8E

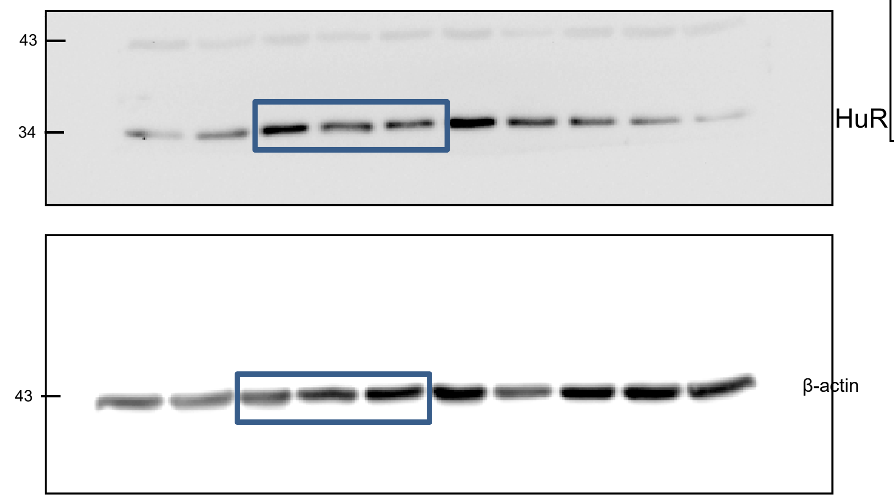

Fig 8E

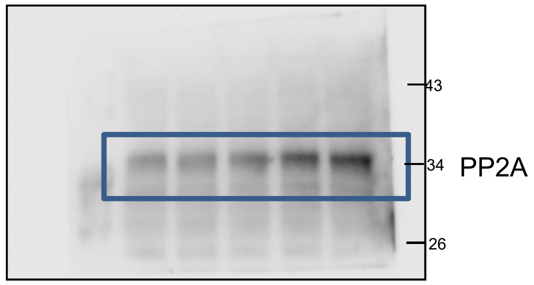

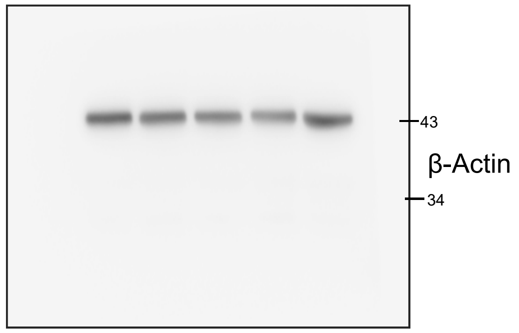

FIG 8 A

- Data 4
- Data 5
- Data 6
- miR146a
- miR125b
- miR16
- Data 10

	A	B	C
	control	Ag83	BHU569
	Y	Y	Y
1	1	1.918528	4.458835
2	1	1.844632	2.918041
3	1	1.863912	3.138336

FIG 8 B

- Data 4
- Data 5
- Data 6
- miR146a
- miR125b
- miR16
- Data 10

	A	B	C
	control	Ag83	BHU569
	Y	Y	Y
1	1.000000	0.632878	0.204476
2	1.000000	0.392292	0.250578
3	1.000000	0.605696	0.540862

FIG 8 C

- miR125b
- miR16
- Data 10
- miR-155
- miR-146a
- miR-125b
- let-7a

	A	B	C
	control	Ag83	BHU569
	Y	Y	Y
1	1.000000	1.604060	2.544983
2	1.000000	1.532725	2.359476
3	1.000000	1.753871	2.952169

FIG 8 F left panel

- Family
- Data Tables
  - HA_HUR_BHU569
  - HA_HUR_BHU569_OKA
  - neo_bhu_oka final
  - HAHUR_OKA_BHU_repeat
  - TNF_Ha_hur-OA
  - IL-1B_HAHUR_OA

	A	B	C	D
	neo-BHU569-OA	neo-BHU569+OA	HA-Hur-BHU569-OA	HA-Hur-BHU569+OA
	Y	Y	Y	Y
1	1	8.046344	1	18.231150
2	1	6.688426	1	28.773460
3	1	7.826316	1	39.487750
4	1	6.758329	1	38.230880

FIG 8 F right panel

Family
Data Tables
HA_HUR_BHU569
HA_HUR_BHU569_OKA
neo_bhu_oka final
HAHUR_OKA_BHU_repeat
TNF_Ha_hur-OA
IL-1B_HAHUR_OA

	A	B	C	D
	neo-BHU569-OA	neo-BHU569+OA	HA-Hur-BHU569-OA	HA-Hur-BHU569+OA
	Y	Y	Y	Y
1	1	2.614738	1	4.993322
2	1	1.140764	1	7.772255
3	1	0.179244	1	4.856780
4			1	3.482202

FIG 8 H left panel

heat killed il 10 level
Data 91
anti tlr 4 PP2A
anti tlr4 TNFa
phospho ago2 lps
pec ago2 associated let7a
oa liver bhu tnfa
oa liver bhu il1b
PP2A LPG
tnfa LPG
oa liver bhu ld count
parasite count oa 2 24 hr

	A	B	C	D
	-OA	+OA	-OA	+OA
	Y	Y	Y	Y
1	1.000000	0.814696	1.565908	2.221879
2	1.000000	0.792793	1.003482	2.569847
3	1.000000	0.627201	0.744322	2.495456
4	1.000000	1.646041	0.769504	7.387059
5	1.000000	1.252580	0.968540	3.254870
6	1.000000	0.879540	1.101250	2.986540

FIG 8 H Right panel

heat killed il 10 level
Data 91
anti tlr 4 PP2A
anti tlr4 TNFa
phospho ago2 lps
pec ago2 associated let7a
oa liver bhu tnfa
oa liver bhu il1b
PP2A LPG
tnfa LPG
oa liver bhu ld count
parasite count oa 2 24 hr

	A	B	C	D
	-OA	+OA	-OA	+OA
	Y	Y	Y	Y
1	1.000000	0.611461	0.429580	2.959515
2	1.000000	1.048747	0.461052	5.939466
3	1.000000	0.990140	1.523127	2.127319
4	1.000000	0.907409	1.633717	2.960466
5	1.000000	1.015487	1.025689	3.125680
6	1.000000	0.895410	1.356890	4.025458

FIG 8 I

heat killed il 10 level
Data 91
anti tlr 4 PP2A
anti tlr4 TNFa
phospho ago2 lps
pec ago2 associated let7a
oa liver bhu tnfa
oa liver bhu il1b
PP2A LPG
tnfa LPG
oa liver bhu ld count
parasite count oa 2 24 hr

	A	B	C	D
	-OA	+OA	-OA	+OA
	Y	Y	Y	Y
1	1.000000	1.005793	0.859757	0.587367
2	0.966160	1.246889	0.863938	0.509917
3	0.798114	0.931525	0.959042	0.487678
4	0.986461	0.978968	0.841479	0.149927
5	0.926374	1.061178	0.962150	0.101321
6	1.000000	1.014428	1.030969	0.108970
